# Circadian rhythmicity persists through the Polar night and midnight sun in Svalbard reindeer

**DOI:** 10.1038/s41598-018-32778-4

**Published:** 2018-09-27

**Authors:** Walter Arnold, Thomas Ruf, Leif Egil Loe, R. Justin Irvine, Erik Ropstad, Vebjørn Veiberg, Steve D. Albon

**Affiliations:** 1 University of Veterinary Medicine, Vienna, Department of Integrative Biology and Evolution, Research Institute of Wildlife Ecology, Vienna, Austria; 2 Norwegian University of Life Sciences, Department of Ecology and Natural Resource Management, Ås, Norway; 3 The James Hutton Institute, Craigiebuckler, Aberdeen, Scotland United Kingdom; 4 Norwegian University of Life Sciences, Faculty of Veterinary Medicine, Oslo, Norway; 5 Norwegian Institute for Nature Research (NINA), Trondheim, Norway

## Abstract

Studies of locomotor activity in Svalbard reindeer reported the temporary absence of diel rhythms under Arctic photic conditions. However, using Lomb-Scargle periodogram analyses with high statistical power we found diel or circadian rhythmicity throughout the entire year in measures of behaviour, temperature in the rumen and heart rate in free-living Svalbard reindeer. Significant diel rhythmicity was only lacking during some of the 15-day intervals analysed in the less frequently measured heart rate. During Polar Night these rhythms were free-running and attenuated. During continual daylight in summer, rhythms where entrained to 24 hours corresponding with the daily variation in the intensity of solar radiation, but attenuated when continuous daylight coincided with the period of growing forage. Diel rhythmicity was reduced during this short period of peak foraging activity, which coincided with peak heart rate and temperature in the rumen, most likely to facilitate fattening when food is abundant. For the rest of the year, heart rate and temperature showed the most pronounced and long-lasting suppression ever found in ungulates. The profound seasonal changes in foraging, metabolic activity, and power of diel and circadian rhythmicity of Svalbard reindeer can be viewed as adaptations to the extreme living conditions in the High Arctic.

## Introduction

Ever since its earliest beginnings, the temporal organisation of life has been shaped by the Earth’s rotation and orbit. No other change in the environment alters the living conditions more, and is more predictable, than the 24 hour light/dark cycle and the annual photoperiod cycle. These cycles are the main drivers in the evolution of endogenous clocks enabling anticipation of the Earth’s periodicities. Circadian clocks are entrained to diel rhythms of 24 hours by external cues (zeitgebers). In polar areas, however, a day/night cycle is absent for much of the year. Therefore, the circadian clock is expected to free-run, i.e. circadian rhythms of activity or physiological functions are expected to show period lengths slightly deviating from 24 hours in the absence of 24-hour zeitgebers^1^.

The Arctic represents a most extreme environment, particularly for endothermic herbivores. Primary production is typically low and restricted to the short summer period when the ground is snow-free and temperatures are above freezing^2^. For most of the year, the weather is much harsher and particularly during mid-winter, when the sun does not rise above the horizon (Polar Night), the ground is frozen, and snow or ice restrict access to food. During the nutritional bottle-neck of winter many herbivorous mammals depend on body fat reserves for energy and insulation^3,4^, which are accumulated during summer. Among ungulates, summer fattening is most pronounced in Arctic species, like reindeer^3^. Therefore, in the Arctic, natural selection may have favoured abandoning diel rhythmicity during the summer period of continual daylight (Midnight Sun) in order to optimally exploit the short time window of plant growth and to maximise energy intake. Indeed, an apparent lack of circadian rhythmicity during seasons of continual darkness or daylight has been reported for a number of Arctic species^5^, and it has been concluded that the loss of circadian rhythmicity may be an ubiquitous trait among resident Polar vertebrates^6^. However, these studies focussed only on the temporal organisation of activity patterns, and did not address the role of food availability.

Here we present results from our long-term study not only of behaviour, but, for the first time, also of physiological variables in Svalbard reindeer (*Rangifer tarandus platyrhynchus*). We demonstrate with high sampling rates the continuous existence of diel rhythmicity not only during periods of day/night cycles but also during continual light, and free-running circadian rhythmicity during Polar Night. However, these rhythms are attenuated by continual darkness in winter and the availability of growing forage in summer.

## Results

### Annual rhythms

**Figure 1:**
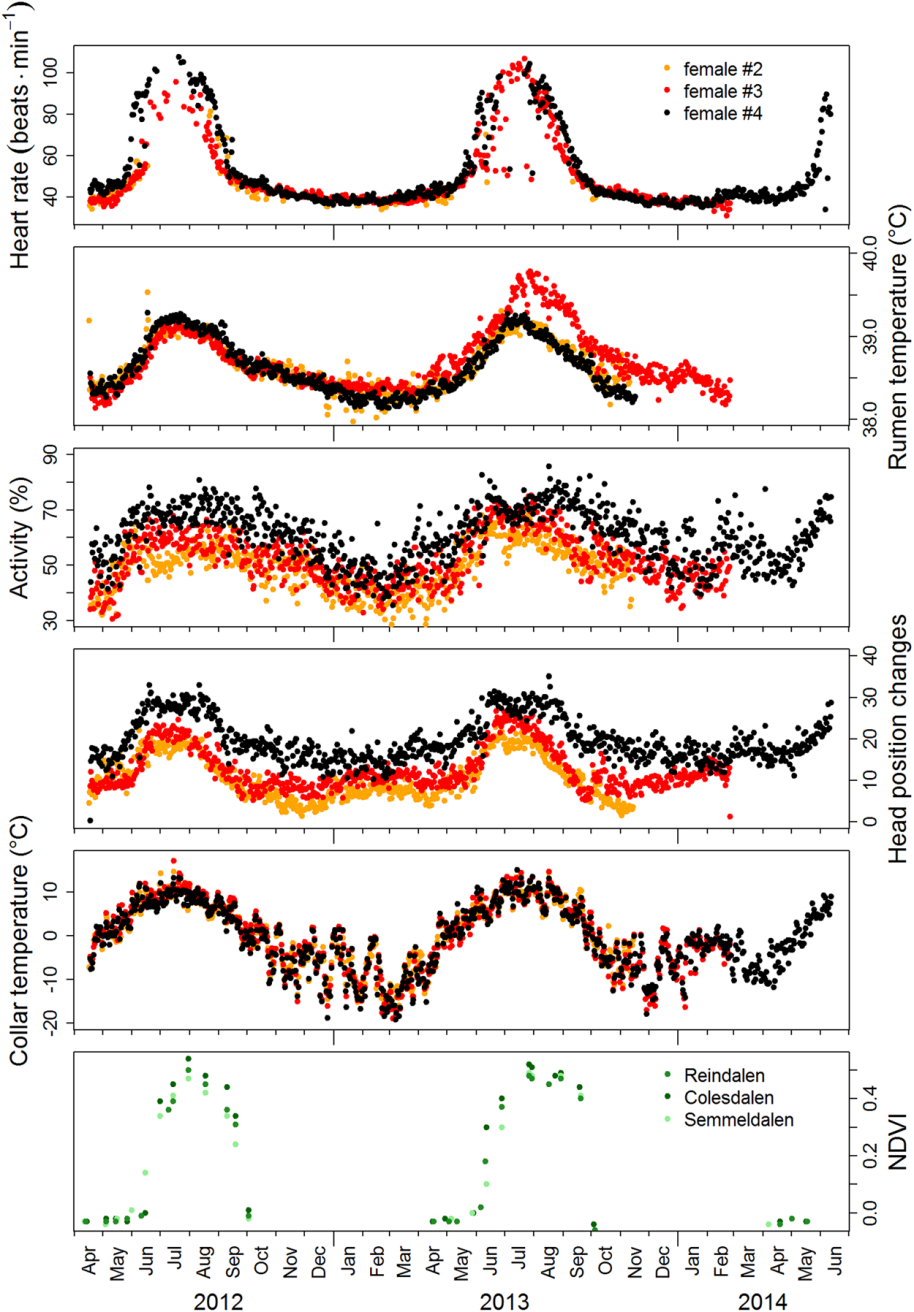
Course of analysed measurements during the study period. Plotted are daily means of telemetrically measured variables of three study individuals represented by differently coloured dots (pregnancy status for each individual in each year is given in Table 1), and 16-day mean NDVI values from three parts of the study area, depicted by variants of green.

**Figure 2:**
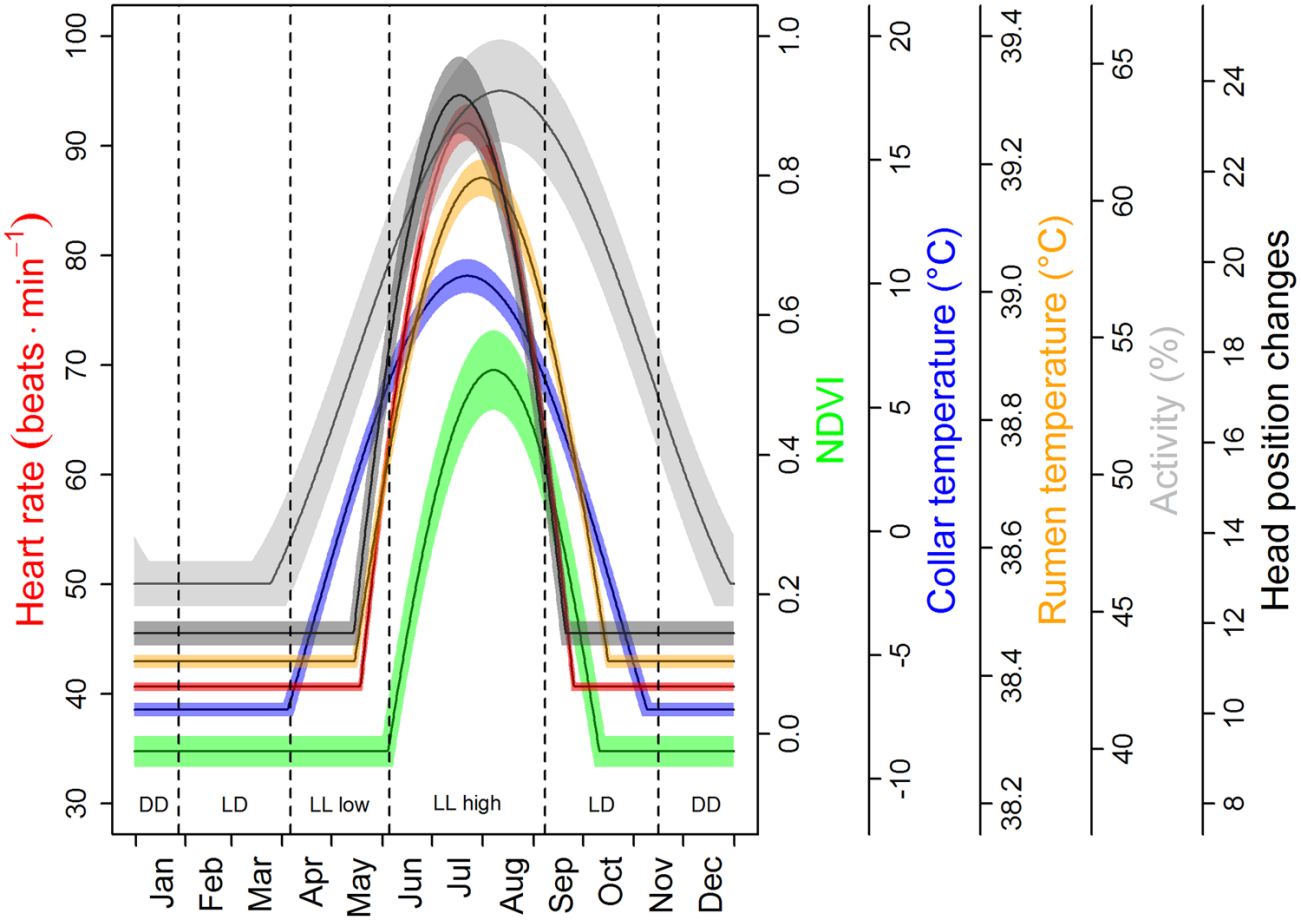
Duration, timing, and height of annual peaks of telemetrically measured variables and NDVI. Sections separated by vertical lines indicate periods with different light and vegetation conditions, i.e. the period when the sun was continuously below the horizon (Polar Night, DD), the periods with daylight and night (LD), the period of continual light (Midnight Sun, LL) before the onset of the vegetation period with NDVI values <0 (LL low), and the period of continual light with above-ground new vascular plant growth (NDVI >0, LL high). Plotted are baseline cosine fits to empirical data shown in Fig. 1, and their 95% confidence intervals (for details see Methods).

Strong seasonality was found in all variables measured, with distinct peaks during the short summer and low values during the rest of the year, with some differences between years and individuals associated with reproductive status (Fig. 1 and Table 1). Daily mean heart rate of animals at rest or moving slowly (‘stationary’ heart rate, HR_s_), more than doubled (126%) within 65 days from mid-May till mid-July from a baseline of ~40 bpm to more than 90 bpm (Fig. 2). In contrast, the increase from the winter baseline through to the summer peak was small for ruminal temperature, T_rumen_ (2%), and moderate for activity (39%), occurring within 77 and 140 days, respectively. The number of vertical head movements during 3-minute measurement intervals increased to the summer peak by 101% within 65 days (Fig. 1).

**Table 1:** Study animals and periods.

Identity	Cohort	2012		2013		2014		2015		Signal duration (months)
		Body mass (kg)	Pregnant Y/N	Body mass (kg)	Pregnant Y/N	Body mass (kg)	Pregnant Y/N	Body mass (kg)	Pregnant Y/N	
# 1	2006	53.5	Y	55.0	Y	57.0	Y	51.0	Y	2
# 2	2004	50.5	Y	NA	Y	54.5	Y	56.0	Y	19
# 3	2006	58.0	N	63.0	Y	51.2	N	60.5	Y	23
# 4	2009	49.0	Y	NA	Y	NA	NA	53.5	Y	26

Details of the four female reindeer given electronic ruminal boli and telemetry collars, showing their cohort (birth year), body mass and whether they were pregnant or not in April each year from 2012 to 2015.

Phase relations of the variables measured were explored by comparing baseline cosine functions fitted to the empirical data (Fig. 2). The onset of the summer period of high HR_s_ (95% confidence interval [CI] May 7–16) coincided with the onset of high T_rumen_ (95% CI May 12–15), and high numbers of vertical head movements (95% CI May 11–16). The end of the period of high HR_s_ (95% CI Sep 23–25) occurred on average 21 days before the end of the period of high T_rumen_ (95% CI Oct 14–17), and 4 days after the period of high numbers of vertical head movements (95% CI Sep 17–22). The onset of the period with high ambient temperature, measured in the collar, T_collar_, (95% CI Apr 1–7) coincided with the beginning of continual light (Apr 5), and ended slightly before the return of continual darkness (95% CI Nov 5–11) on Nov 15. However, activity began to increase from the winter low by mid-March (95% CI Mar 12-Apr 1), a few days before T_collar_ began to rise, and remained above the winter baseline until Dec 19-Jan 8 (95% CI), i.e., way into the period of continual darkness and lowest T_collar_.

### Daily and circadian rhythms

**Figure 3:**
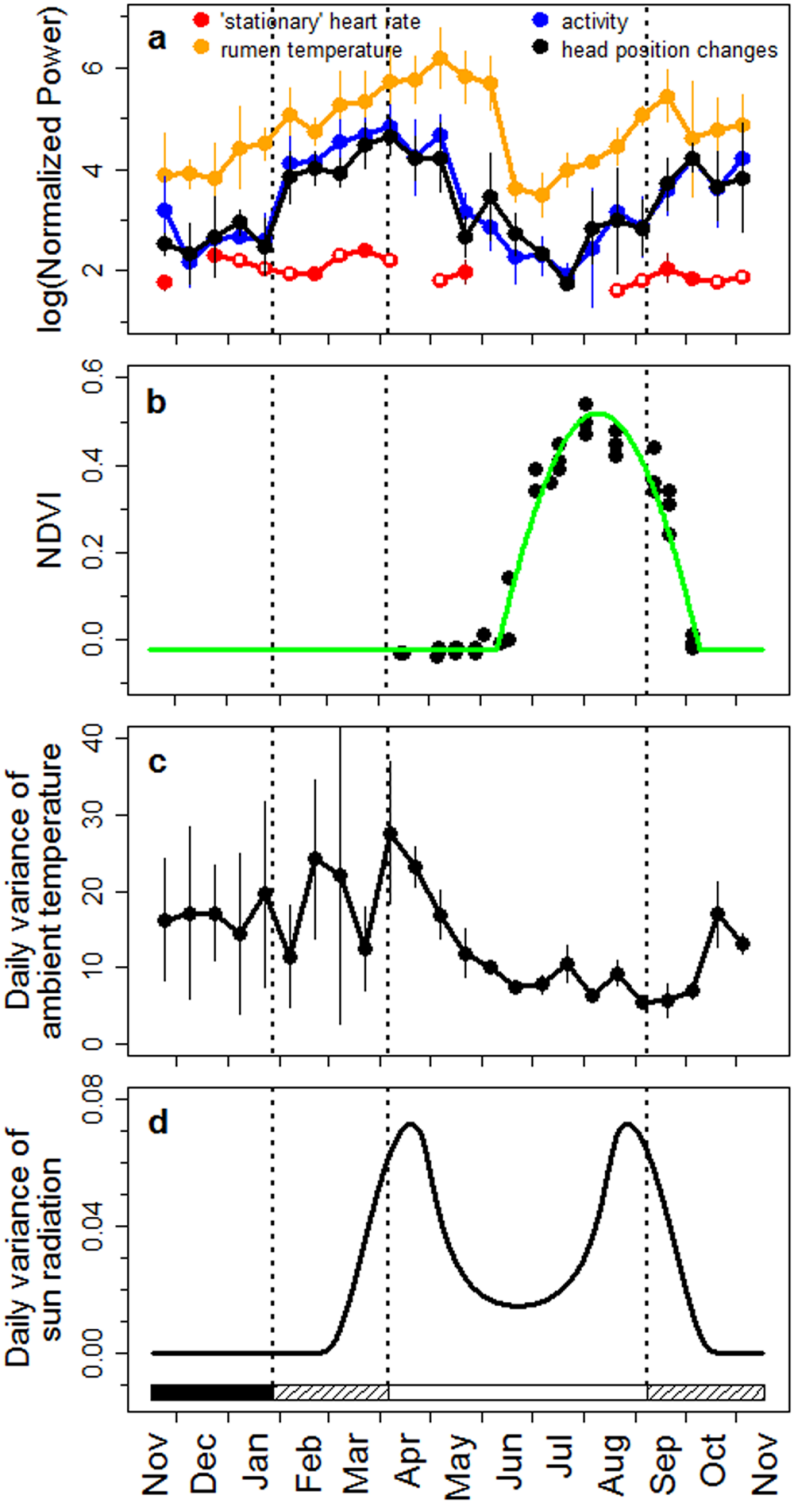
Annual course of circadian rhythmicity and potential zeitgebers. (**a**) Average normalized power of significant rhythms (p < 0.05) with period lengths between 21 and 27 hours, calculated with Lomb-Scargle periodogram analyses for 15-day intervals from Nov 15, 2012 to Nov 15, 2013, for ‘stationary’ heart rate (red), rumen temperature (orange), activity (blue), and head position changes (black; means and 95% confidence intervals reflecting variation between individuals, open symbols are single values). Note that more significant rhythms are found here compared to the analyses depicted in third panels of Supplementary Figs S4–S14. This is because the probability of detecting significant peaks depends on the number of periods scanned, i.e., it is higher when searching for periods between 21 to 27 hours than between 0.5 to 30 hours, as done for Supplementary Figs S4–S14. (**b**) 16-day averages of NDVI values from the three major parts of the study site for 2013 (see Fig. 1) and a baseline cosine fit to this data. (**c**) Ambient temperature measured in the collar (15-day means of daily variation and 95% confidence intervals, reflecting variation between individuals). (**d**) Daily variance of direct maximum solar radiation at the study site (disregarding weather effects). Horizontal bars and dotted vertical lines in each plot indicate periods of continual darkness (black), daylight/night changes (hatched), and continual daylight (open). Note that periods of daylight/night changes begin before and end after the sun firstly and lastly arises above the horizon. This difference results from using civil twilight for calculating beginning and end of daylight.

**Figure 4:**
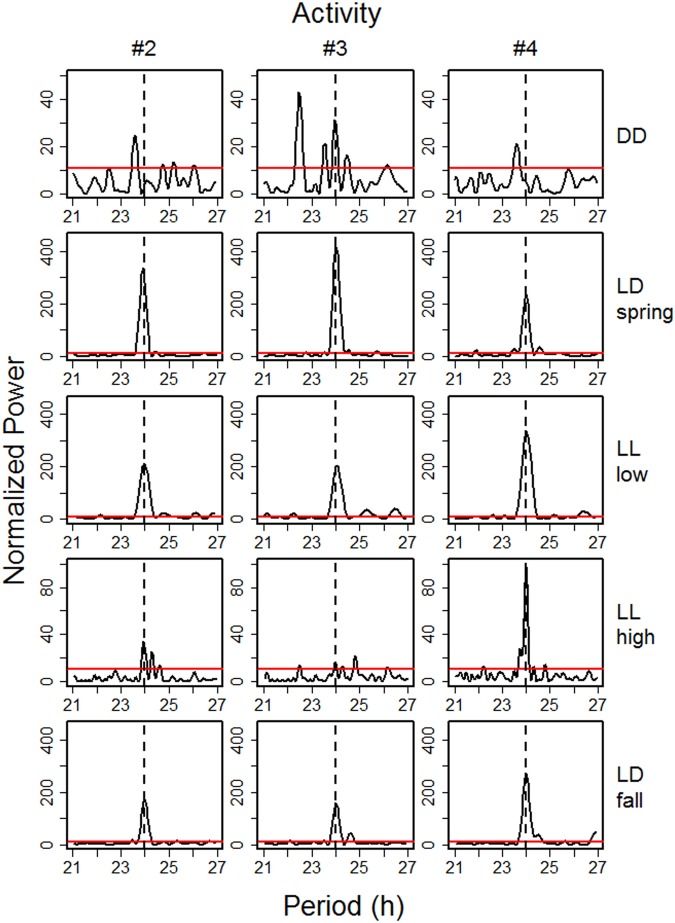
Comparison of rhythms of activity with period lengths between 21 and 27 hours of study animals #2, #3, and #4 between Nov 15, 2012 and Nov 15, 2013. Plotted are normalized powers of rhythms of activity. Each row of panels represents the different time periods: Polar Night (DD), daylight and night (LD), continual light (Midnight Sun, LL) before the onset of new vegetation growth with NDVI values <0 (LL low), and continual light with above-ground new vascular plant growth (NDVI >0, LL high). Horizontal red lines indicate the significance threshold of p = 0.001.

**Figure 5:**
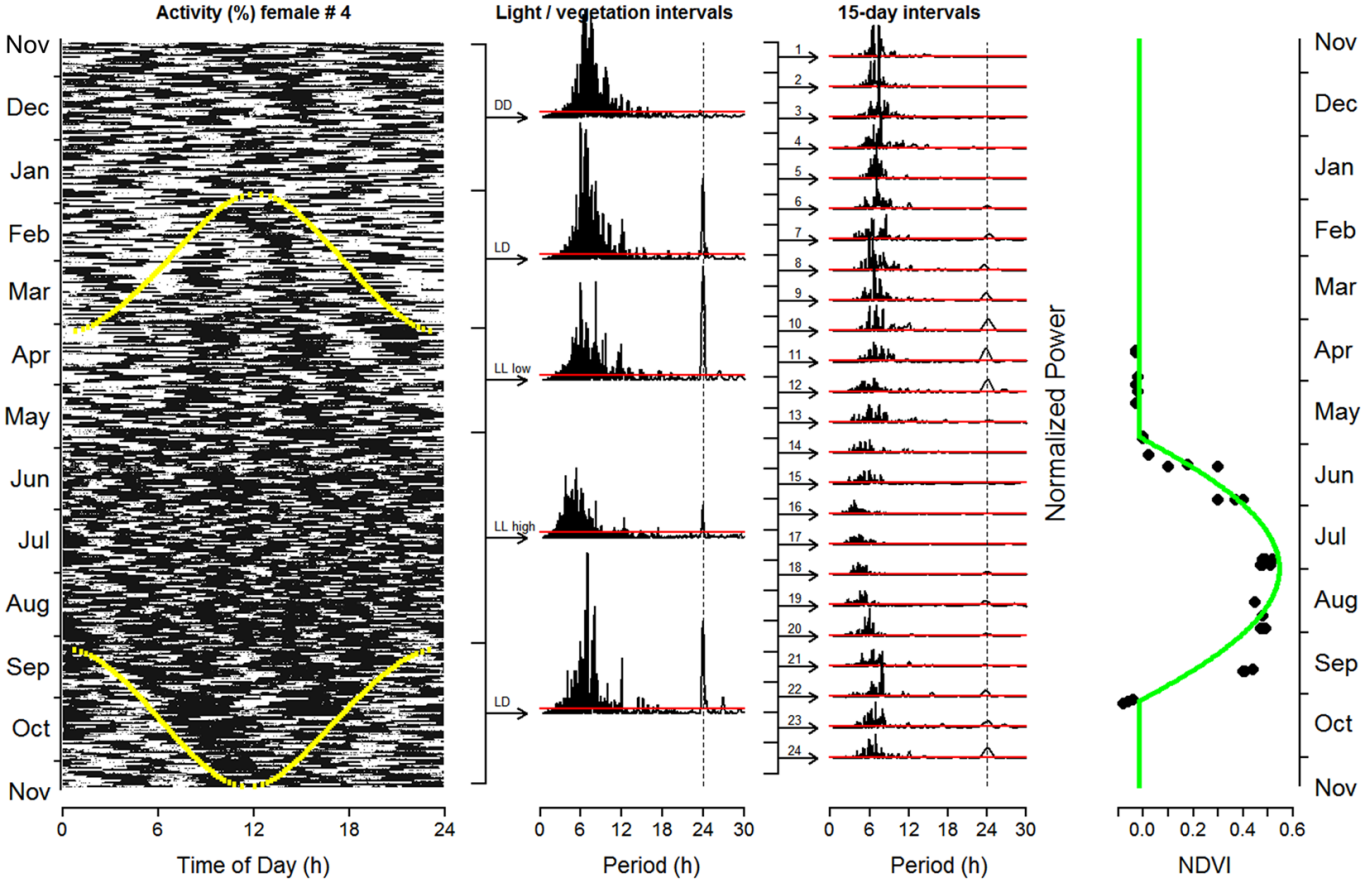
Daily patterns of activity of individual #4 between Nov 15, 2012 and Nov 15, 2013 as a representative example. Leftmost panel: Actogram of activity showing black bars for each 3 minute interval, with a height proportional to activity, one horizontal line for each day. Yellow lines indicate beginning and end of civil twilight (i.e. the geometric centre of the Sun’s disk is ≤6° below the horizon), a luminance that is sufficient to cause photic responses, e.g., suppression of melatonin production^45^. Second panel: Normalized power of rhythms with period lengths between 30 minutes and 30 hours identified by Lomb-Scargle periodogram analysis. Horizontal red lines indicate the significance threshold of p = 0.001. Lines and arrows to the left indicate the intervals analysed, i.e. the period when the sun is continuously below the horizon during winter (Polar Night, DD), the periods with daylight and night (LD), the period of continual light (Midnight Sun, LL) before the onset of the vegetation period with NDVI values <0 (LL low), and the period of continual light with above-ground live vascular plants (NDVI >0, LL high). Third panel: as second panel but with periodograms of 15-day intervals. Rightmost panel: 16-day mean NDVI values from the three major parts of the study site for 2013 (see Fig. 1) and a baseline cosine fit to this data.

Rhythms with a period length (τ) of ~24 h, causing significant periodogram peaks at p < 0.05, were found throughout the year and in all variables measured (Figs 3a and 4, Supplementary Figs S1–S3). Only for HR_s_, which was less frequently measured, were no significant rhythms in the 21–27 h range detectable in some of the 15-day intervals analysed (Fig. 3a). In order to discriminate between the influences of continual daylight and food availability on the rhythmicity of physiological and behavioural variables, the period from April 5 to September 7 (Midnight Sun) was divided into two parts according to the Normalized Difference Vegetation Index (NDVI, Fig. 2). During the first part, to early June, food availability resembled that typical for winter (NDVI values <0), whereas during the second part from early-June to early-September, NDVI values >0 indicated growing vegetation (Fig. 2). During the first part of the period with continual daylight but little or no new plant growth, clear 24 hour rhythmicity was present in both behavioural and physiological measurements (Figs 3a, 4 and 5, Supplementary Figs S1–S14), in the case of T_rumen_ even with the highest normalized power (Fig. 3a and Supplementary Fig. S2). Diel rhythmicity persisted but became weaker during the second part, when most vegetation growth occurred, and when the daily variance of solar radiation was lowest (Figs 3, 4 and 5, Supplementary Figs S1–S14). With respect to entrainment, HR_s_ was again an exception with apparently low or lacking entrainment to 24 hours during Midnight Sun (Fig. 3a and Supplementary Fig. S3). The daily variation of solar radiation, peaking at begin and end of the period of Midnight Sun (Fig. 3d), was an important predictor of the power of diel rhythmicity, as were high values of NDVI for its attenuation during the period of continuous daylight (Table 2).

**Table 2:** The influence of environmental cues on diel rhythmicity of behaviour and physiology during the course of the year; results from linear mixed modelling.

Predictor^b^	Response^a^											
	Activity			Head position changes			Rumen temperature			Heart rate		
	beta^c^	F_(1,63)_	p	beta^c^	F_(1,61)_	p	beta^c^	F_(1,66)_	p	beta^c^	F_(1,19)_	p
Daily variance of sun radiation	0.531	22.97	<**0**.**001**	0.537	21.35	<**0**.**001**	0.713	50.22	<**0**.**001**	0.371	1.77	0.199
Daily variance of ambient temperature (T_collar_)	0.022	0.04	0.843	−0.047	0.16	0.686	−0.120	1.37	0.246	0.140	0.59	0.453
Average NDVI-value (from baseline-cosine fit)	−0.676	26.60	<**0**.**001**	−0.670	24.11	<**0**.**001**	−0.695	36.50	<**0**.**001**	−0.656	2.96	0.101

^a^Response variables are logarithmic transformed powers of circadian rhythms of behaviour and physiology detected with Lomb-Scargle periodogram analyses during subsequent time intervals of 15 days with period lengths between 21 and 27 hours (Fig. 3a). To account for repeated measurements of individuals, we included individual ID as a random factor in the model.

^b^Predictors are daily variation of direct maximum solar radiation at the study site (disregarding weather effects), mean daily variation of ambient temperature during respective 15-day intervals (measured in the collar), and 16-day means of NDVI-values determined from baseline-cosine fit (Fig. 3b–d).

^c^Standardized regression coefficient.

Significant p-values are presented in bold font.

As expected, when reindeer were exposed to daily light/dark cycles (end of January to early April, early September to mid-November, see first panel in Fig. 5), all physiological and behavioural variables measured showed strong diel rhythmicity with a τ of 24 hours (Figs 3a and 4, Supplementary Figs S1–S3). During the period of Polar Night, temporal organisation of physiological and behavioural variables remained rhythmic, but with lower normalized power, similar to rhythms found during the second part of the period with continual daylight and high values of NDVI (Fig. 3a). However, in contrast to the period of Midnight Sun, during the Polar Night the significant peaks almost always had a τ ≠ 24 hours (Fig. 4, Supplementary Figs S1–S3).

### Ultradian rhythms

Ultradian rhythms with τ between ~4–8 hours were present throughout the year with strong normalized power in locomotor activity and changes of head positions during three-minute measurement intervals. During winter the dominant period of these rhythms was ~6 hours and shortened to ~4 hours during the summer period (Fig. 5, Supplementary Figs S4–S8). In contrast, ultradian rhythmicity was less pronounced in T_rumen_ and HR_s_ (Supplementary Figs S9–S14).

## Discussion

**Figure 6:**
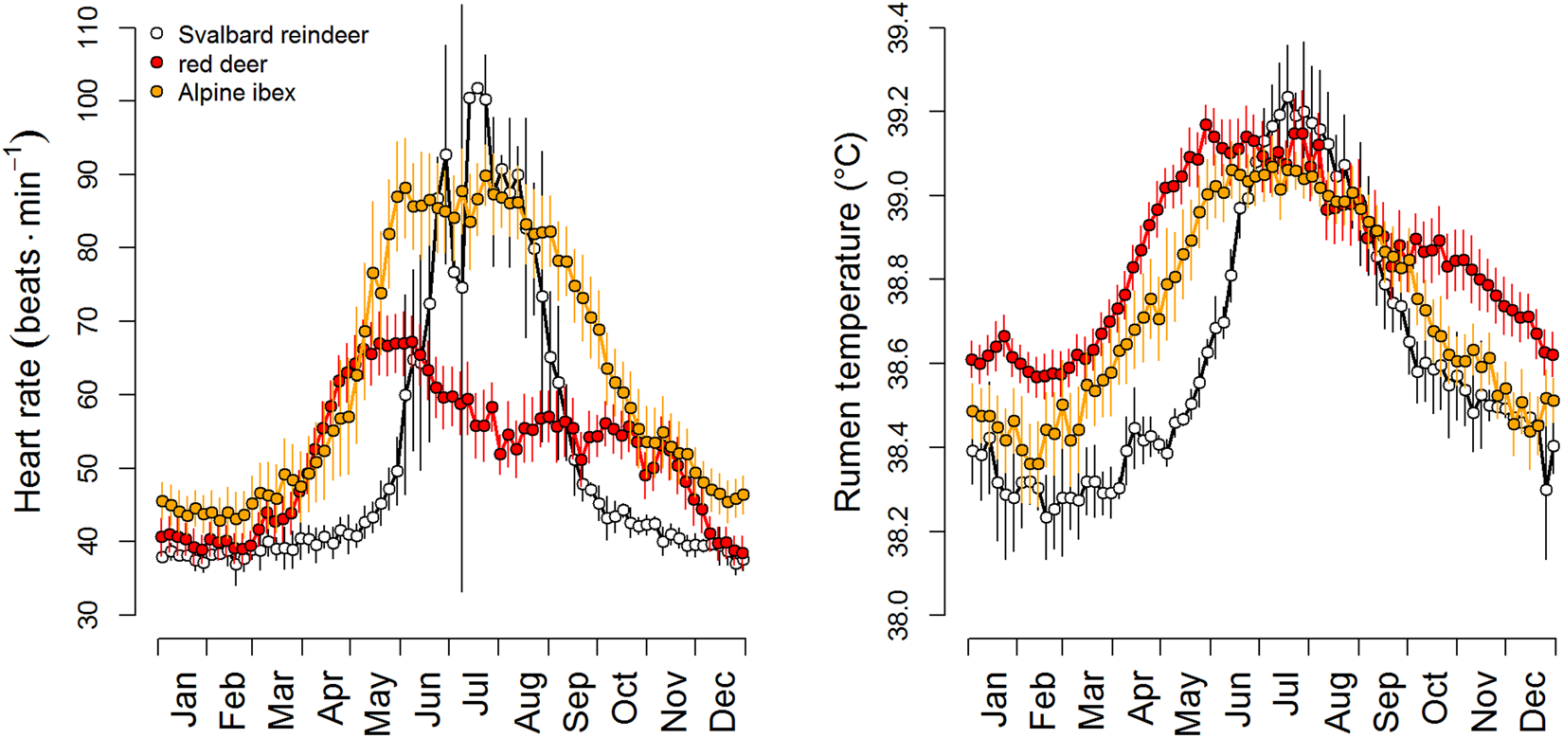
Comparison of the annual cycles of three species of ungulates dwelling in seasonal environments. Left, stationary heart rates; right, rumen temperatures of the study animals (white symbols), red deer (red symbols) redrawn from^13^, and Alpine ibex (orange symbols) redrawn from^12^. Points are means over periods of 5 days. Error bars are 95% confidence intervals of means and reflect variation between individuals.

Our results clearly demonstrate that rhythmicity with τ of ~24 h persists throughout the year in Svalbard reindeer. This is at odds with previous reports of an apparent lack of such rhythmicity in this species in the absence of a day/night cycle^6–8^. However, the power of this rhythmicity diminished considerably during Polar Night and a τ of typicall y ≠ 24 hours indicated free-running endogenous circadian rhythmicity. Conversely, rhythms remained well entrained during continual light conditions in summer, except for HR_s_, and had a high power before the onset of the plant growing season (Fig. 3a,b). Diel rhythmicity diminished later reflecting the need for intense foraging all around the clock when forage availability and quality was at its best. The attenuation of rhythmicity also coincided with minimal daily variance in solar radiation. However, continual light, i.e. a loss of zeitgeber, is unlikely to be solely responsible for the attenuation of rhythmicity during June to August, because we found no indication of free-running rhythms as in winter. Therefore, we attribute the apparent diminution of daily rhythms to foraging requirements. However, even then diel rhythmicity persisted as can be seen particularly in the 24 hour rhythmicity of T_rumen_ (Fig. 3a and Supplementary Fig. S2). The summer period with high values of HR_s_, T_rumen_, and locomotor activity in Svalbard reindeer is the shortest ever found in ungulates so far (Fig. 6). Furthermore, the difference of HR_s_ and T_rumen_ between winter and summer is the most pronounced among the species where this has been measured^9–13^. Presumably the extremely short time-window during which plant growth is sufficient for fattening requires Svalbard reindeer to attenuate diel rhythmicity and feed throughout 24 hours, thereby largely ignoring their circadian master clock, even though this is still running.

The spring increase of locomotor activity clearly preceded that of physiological variables and vertical movements of the head, i.e. food intake. Also, it preceded the spring increase of collar temperature (T_collar_), although low ambient temperatures basically reduced locomotor activity. Similarly, activity remained high until around the winter solstice, despite T_collar_ being close to the winter low from the end of November onwards. The reason for the much wider peak of activity compared to other variables remains enigmatic. Seasonal migration would be a possibility, but can be excluded for Svalbard reindeer^14^. Although severe winter weather, particularly ‘icing events’ associated with ‘rain-on-snow’, may cause local animal movement (5–10 km) to seek accessible forage^15,16^, Svalbard reindeer are regarded as basically sedentary^14^. Populations separated by as little as 40–50 km are genetically distinct^17^.

The frequency of vertical movements of the head increased about two weeks before the increase in NDVI. The food ingested during these two weeks will have been the standing crop from the previous summer with some new shoots of plants growing on ridge communities, where snow melt is earliest^2^. Therefore, the marked increase of foraging activity before substantial new growth of plants became available, suggests photoperiodic control of appetite, as described for many other ungulates^13,18–20^.

Our results indicate the continuous function of the circadian clock throughout the year in Svalbard reindeer, but the animals barely adhere to it under certain circumstances. During the period of Midnight Sun, entrainment to a 24 hour period was evident, despite continual daylight. In the absence of a day/night cycle changes in ambient temperature, which can entrain the circadian clock in rodents, e.g.^21^, did not act as alternative zeitgebers (Table 2). Possibly, the cyclic diel changes of solar radiation (Fig. 3d and Table 2), or associated changes of luminance or skylight colour^22–24^ may act as zeitgebers. However, these cues failed to entrain circadian rhythms in another Arctic species, the Lapland Longspur^25,26^, that shows persistent daily rhythms in activity and melatonin production und continuous illumination in summer.

During the Polar Night these potential alternative zeitgebers were absent, at least around the winter solstice. As a result, circadian rhythms were free-running with τ deviating from 24 hours and with different τ for HR_s_, T_rumen_ and behavioural measures within individuals (Fig. 5, Supplementary Figs S1–S3), suggesting internal desynchronization of rhythms^27,28^. Multiple significant peaks during the Polar Night with τ of ~24 h indicate different but fairly stable τ across the mid-winter months. Such shifts of individual τ are well known, for example as ‘after effects’, typically occurring when an organism is transferred from an environment with distinct zeitgebers into one without^29^. Our findings support the view by Williams *et al*.^5^ that persistent circadian rhythms in continual darkness may be adaptive due to interdependence between circadian clock function and homeostatic processes.

In line with previous reports^6–8^, we found strong and similar ultradian rhythms in activity and vertical movements of the head, with seasonally changing period lengths. Shorter period lengths were present during peak vegetation growth and probably indicate more frequent fills of the gut, greater digestibility and hence shorter gut passage time when food is abundant and of high quality^6,20,30^.

The reason for the discrepancy between our results and previous reports seems to be partly due to a difference in statistical power resulting from both sample size and type of periodogram analysis applied. Loe *et al*.^8^ recorded hourly activity only every 5^th^ day and created time series by merging these measurement days. This lack of continuous monitoring presumably prevented the detection of weak diel rhythmicity. We also failed to find significant diel rhythmicity throughout continual light or darkness, presumably due to insufficient sample size, in the less frequently measured HR_s_ (every 21 minutes compared to 3 minutes for activity, and only if the animal was at rest or moving slowly) in individual #2 (Supplementary Fig. 3), or in some 15-day intervals (Fig. 3a). Van Oort *et al*.^6,7^ on the other hand, recorded activity at 10–15 minute intervals but evaluated their data with F-periodogram analysis. The algorithm underlying this method is actually mathematically equivalent to the Chi^2^-periodogram^31^, and this method is known to have poor power to detect existing rhythms compared with the Lomb-Scargle periodogram^32^. We used the Lomb-Scargle periodogram because this method is particularly suited to detect periodic components in unequally sampled time-series and data sets with missing values, but restricts all calculations to actually measured values. Therefore, Lomb-Scargle periodogram analysis is superior to other statistical methods applied in biomedical rhythm research, including Fourier analysis e.g.^8^, if data are obtained over longer periods of time which typically have such features^32^.

Taken together, our data suggest that the circadian master clock in reindeer continues to function throughout the year, but daily cycles are easy to detect only in variables with a high signal to noise ratio, such as T_rumen_, and if data are sampled frequently. However, these results do not rule out that certain other diurnal rhythms in reindeer, such as the production of melatonin, are not governed by the circadian system as typical for rodents, but by acute responses to the light/dark cycles^33^. This type of regulation of the melatonin rhythm, which has been demonstrated in captive reindeer^33^, may well represent an adaption to a weak output of the circadian axis under certain photic conditions, but is not evidence for a complete absence of circadian clock signals.

To summarize, the length of the metabolically intense summer phase of Svalbard reindeer is the shortest found in ungulates so far, and the differences between summer and winter in physiology and behaviour are the highest ever reported. Diel rhythmicity persists throughout the year, although attenuated by continual darkness and high availability of food. During these periods, temporal organisation of behaviour and physiology was to a large degree shaped by ultradian rhythms of foraging behaviour.

## Methods

### Study area and climate

The research was carried out in Nordenskiöldland, Svalbard (77°50′–78°20′N, 15°00′–17°30′E). Our main study area was centred in Colesdalen, Semmeldalen, and parts of Reindalen, and their side-valleys. The generally wide, U-shaped valleys are mostly vegetated (up to about 250 m altitude), though the short growing season means that above-ground live vascular plant biomass in vegetated habitats averages only 35 g per m^2^ (annual range 23–46 g per m^2^)^2^. Nonetheless, the area supports a relatively high density of reindeer compared to other parts of Svalbard^34^ with the population almost doubling over two decades^35^. The lower-lying, wetter, and more productive, pastures are grazed during summer, but in winter forage tends to be less accessible here because of deep or hard snow, or ice. Therefore, in winter reindeer tend to feed on wind-blown vegetated ridges, and at higher elevations see also^36^.

Direct maximum solar radiation at the study site as a function of the day of year and time of day (disregarding weather effects), was computed using equations given at www.pveducation.org. These computations involve calculating the sun’s zenith angle, for which we used function SZA from the R package RAtmosphere^37^.

### Remote-sensed vegetation indices

We used remote-sensed values of the Normalized Difference Vegetation Index (NDVI) between April 2012 and May 2014 for the valleys Colesdalen, Semmeldalen, and Reindalen in our study area. The NDVI values from the MODIS Terra platform are based on 16-day composites on a spatial resolution of ~250 m available from (https://search.earthdata.nasa.gov), and are above zero from the start of snow melt in June until October when failing light levels limit the records.

### Telemetry system

The four animals sampled in this study were instrumented with GPS-collars (Vectronic Aerospace) at time of capture, which were modified by the Research Institute of Wildlife Ecology. The modification consisted of adding two components, a cylindrical ruminal unit (22 × 80 mm, 100 g) to record physiological measurements, and a unit situated in one of the collar’s battery slots, used for communication with the ruminal unit, data storage and for measuring activity. Activity of the animals and vertical movements of the head were detected in the collar unit with a tri-axial vibration switch, and a mercury switch, respectively. Activation of these switches was checked every second. The proportion of activations of the tri-axial vibration switch during a 3-minute measurement interval was our measure of locomotor activity. The mercury switch was activated by tilting the perpendicular position of the collar by more than approximately 15 °C. The number of activations of the mercury switch (head up/down movements) during a 3-minute measurement interval was our indicator of grazing activity. Lastly, a temperature sensor in the black instrument and battery case of the collar, located under the animals’ head, measured ambient temperature (T_collar_). Numbers of activations of the tri-axial vibration switch, of the head-movement mercury switch, and mean T_collar_ during 3-minute measurement intervals were stored in the collars’ solid–state memory.

The ruminal unit was introduced into the pharynx with an applicator and then swallowed by the reindeer into the reticulum. Temperature in the rumen (T_rumen_) was measured every 3 minutes by a thermistor in the ruminal unit, calibrated in a water bath before administration at 5 °C-increments between 20–40 °C with an accuracy of 0.1 °C. T_rumen_ measurements contained brief periods of rapid cooling because of ingested cold water, snow or food. We discarded these data from analyses by removing T_rumen_ measurements when T_rumen_ changed between subsequent 3-minute measurement intervals by more than 0.25 °C. An integrated bi-axial acceleration sensor in the unit detected vibrations caused by the beating of the heart. Heart rate was calculated from the time intervals between two accelerations above an adaptive trigger level for measurement periods of 3 minutes. To extend battery life, the next 3-minute period of heart rate measurement was made after a pause of 18 minutes. Hence heart rate data were collected at 21-minute intervals. To save energy, the acceleration sensor was inactivated for 0.5 seconds upon acceleration above the trigger level. Therefore, the upper detectable limit of heart rate measurements was 120 beats per minute. We considered this limit as biologically meaningful because a higher heart rate is very unlikely to occur in a reindeer at rest or slow movement such as grazing. Data from the ruminal unit were sent *via* UHF-link to the collar unit and stored there in solid–state memory. For further details about the technology, see^38^.

Apart from contractions of the heart, movements of the animal and reticulum contractions also activated the acceleration sensor of the ruminal unit. Further, the UHF signal used for communication between the ruminal and collar unit was sometimes disturbed. Therefore, false measurements were removed from raw data after download with a stepwise software filter as follows:

Step 1: Removal of intervals with too few detectable heart beats. If the sum of time periods between accelerations above trigger level added up to less than 70% of 3 minutes, this 3-minute measurement interval of heart rate was discarded.

Step 2: Removal of data during locomotor activity. Activity was classified from the output of the triaxial vibration switch in the collar for each individual over the entire period of deployment of a telemetry system. The distribution of this output was always bimodal, typically with peaks very close to 0% and 100%. The lowest point of the distribution between the two modes was used as cut-off to classify each 3-minute measurement interval as spent active or inactive. All 3-minute measurement intervals of heart rate from active animals were discarded.

Step 3: Determination of the modal heart rate. For each of the remaining 3-minute measurement intervals, a Kernel density function was computed for the stored heart rate values, and the heart rate at maximum density was taken as the modal heart rate for this particular 3-minute interval.

Step 4: Removal of outliers. A spline curve (using function ‘gam’ in R) was fitted to the filtered heart rate data sampled over the entire period of deployment of a telemetry system. If the difference between values predicted from the spline fit (i.e. long-term trends in the data) and the modal heart rate within each 3-minute interval exceeded +30 or −20, this heart rate value was discarded.

The remaining data represent heart rate of animals at rest or moving slowly and we therefore refer to these filtered data as the ‘stationary’ heart rate (HR_s_)^12,13^.

### Focal reindeer

In April 2012 four female reindeer from our marked population of ca. 400 individuals were caught by net from snowmobiles, measured, weighed to the nearest 0.5 kg^38,39^, and instrumented with the telemetry system (Table 1). Two of these four individuals (#1 and #3, both from the 2006 cohort) were re-captured the following April 2013. In April 2014 both these animals, together with #2 (2004 cohort) were re-captured and the collars were removed. Individual #4 (2009 cohort) was again missed but finally re-captured in April 2015 (see Table 1). Body mass is not available for years when individuals were not recaptured (NA in Table 1). Pregnancy status was assessed by ultrasound scans upon capture. Individuals #2 and #4 were observed during early August 2013 each with a calf on the heel. All procedures performed in this study were in accordance with relevant Norwegian regulations and guidelines. All capture and live animal handling procedures were performed under licences from the Norwegian Food Safety Authority and its predecessor the Norwegian Animal Research Authority (FOTS ID: 8417).

### Quantification and statistical analysis

Raw data were downloaded from collars after retrieval. Obvious outliers beyond expected physiological limits typically occurred immediately after deploying telemetry devices and during periods before batteries died. Furthermore, T_rumen_ of female #4 showed an unphysiological continuous decline after mid-November 2013, apparently due to a failure of the temperature sensor of the ruminal unit. These data were removed before applying the aforementioned filtering routines.

The beginning and end of summer periods with higher values were determined by fitting a special periodic regression model to empirical data, the so-called ‘baseline cosine function’. The baseline cosine function is an appropriate model to analyse rhythms that show temporal peaks above an otherwise largely stable baseline^40^. The parameters baseline, fraction of a cosine curve added to baseline to describe the peak, height of the sinusoidal peak above baseline, and acrophase of the rhythm were estimated for all variables measured with the R-function ‘nls’; 95% confidence intervals of the parameter estimates were determined with R-package ‘nlstools’^41,42^ to draw 95% confidence belts of the fits. Importantly, the function only delivers a baseline if its inclusion improves the fit compared to a complete cosine function^40^.

Searches for periodicities with period lengths between 30 minutes and 30 hours (to search for ultradian, diel and circadian rhythms), or 21 to 27 hours (to focus on diel and circadian rhythms), respectively, were performed with Lomb-Scargle periodogram analyses R-package ‘lomb’^32^. This method is superior to other statistical methods applied in biomedical rhythm research, including Fourier analysis, because it is particularly suited to detect periodic components in unequally sampled time-series, which includes data sets with missing values, but restricts all calculations to actually measured values^32^. Data obtained with loggers over longer periods of time typically have such features. For the Lomb-Scargle periodogram analyses we discarded data from individual #1 because the two months of valid measurements were too short.

We performed Lomb-Scargle periodogram analyses separately for periods with different light and vegetation conditions, i.e. the period when the sun is continuously below the horizon during winter (DD), the periods with daylight and night (LD), the period of continual light before the onset of the vegetation period with NDVI values <0 (LL low), and the period of continual light with above-ground live vascular plants (NDVI >0, LL high). Although we are aware that neither the Polar Night nor the Midnight Sun are conditions of truly constant illumination, we simply use the labels “DD” and “LL”, respectively, to enhance readability and retain standard chronobiologic terminology. For visualizing changes in periodicities over the year on a smaller scale, we further performed Lomb-Scargle periodogram analyses for subsequent intervals of 15 days, which exceeds the recommended minimum duration of ten periods for biological rhythm analyses^31^. The Lomb-Scargle periodogram provides the normalized power of each of the periods inspected. The term normalized refers to the fact that the raw power (i.e. the goodness-of-fit of the best period) is divided by twice the total variance in the time series (equation 1 in^32^). This normalization ensures that, in the case of the null hypothesis, the power follows an exponential distribution, which allows one to test whether the periodogram peak exceeds the level of statistical significance^43^. The probability of detecting a significant peak decreases with an increasing number of periods inspected, because this increases the probability of rhythms occurring by chance. Prior to computing periodograms, linear trends were removed from the time series.

The influence of environmental cues on diel rhythmicity of behaviour and physiology during the course of the year was tested with linear mixed modelling (R-package ‘nlme’^44^). We accounted for repeated measures by including individual identity as a random intercept factor into the models.

## Electronic supplementary material

Supplementary Information

## Data Availability

The datasets generated and analyzed during the current study are available at Dryad Digital Repository http://datadryad.org/.
