# Supplementary material for: Circadian rhythmicity persists through the Polar night and midnight sun in Svalbard reindeer
Source: Sci Rep. 2018 Sep 27;8:14466. doi: 10.1038/s41598-018-32778-4 (PMC6160466; doi:10.1038/s41598-018-32778-4)
Supplement: Supplementary file 1 — Supplementary Information [file 41598_2018_32778_MOESM1_ESM.pdf]

Circadian rhythmicity persists through the Polar night and midnight sun in Svalbard reindeer+

Walter Arnold [1*], Thomas Ruf [1], Leif Egil Loe [2], R. Justin Irvine [3], Erik Ropstad [4], Vebjørn Veiberg [5] and Steve D. Albon [3]

[1] University of Veterinary Medicine, Vienna, Department of Integrative Biology and Evolution, Research Institute of Wildlife Ecology, Austria

[2] Norwegian University of Life Sciences, Department of Ecology and Natural Resource Management, Norway

[3] The James Hutton Institute, Craigiebuckler, Aberdeen, Scotland, United Kingdom

[4] Norwegian University of Life Sciences, Faculty of Veterinary Medicine, Oslo, Norway

[5] Norwegian Institute for Nature Research (NINA), Trondheim, Norway

*Corresponding Author:

Walter Arnold
University of Veterinary Medicine, Vienna, Department of Integrative Biology and Evolution, Research Institute of Wildlife Ecology
Savoyenstr. 1, 1160 Vienna, Austria

E-mail: walter.arnold@vetmeduni.ac.at
Phone: +43-1-25077-7100
Fax: +43-1-25077-94-7100

+Dedication in memoriam Prof. Dr. Serge Daan (1940-2018), one of the greatest thinkers in Chronobiology

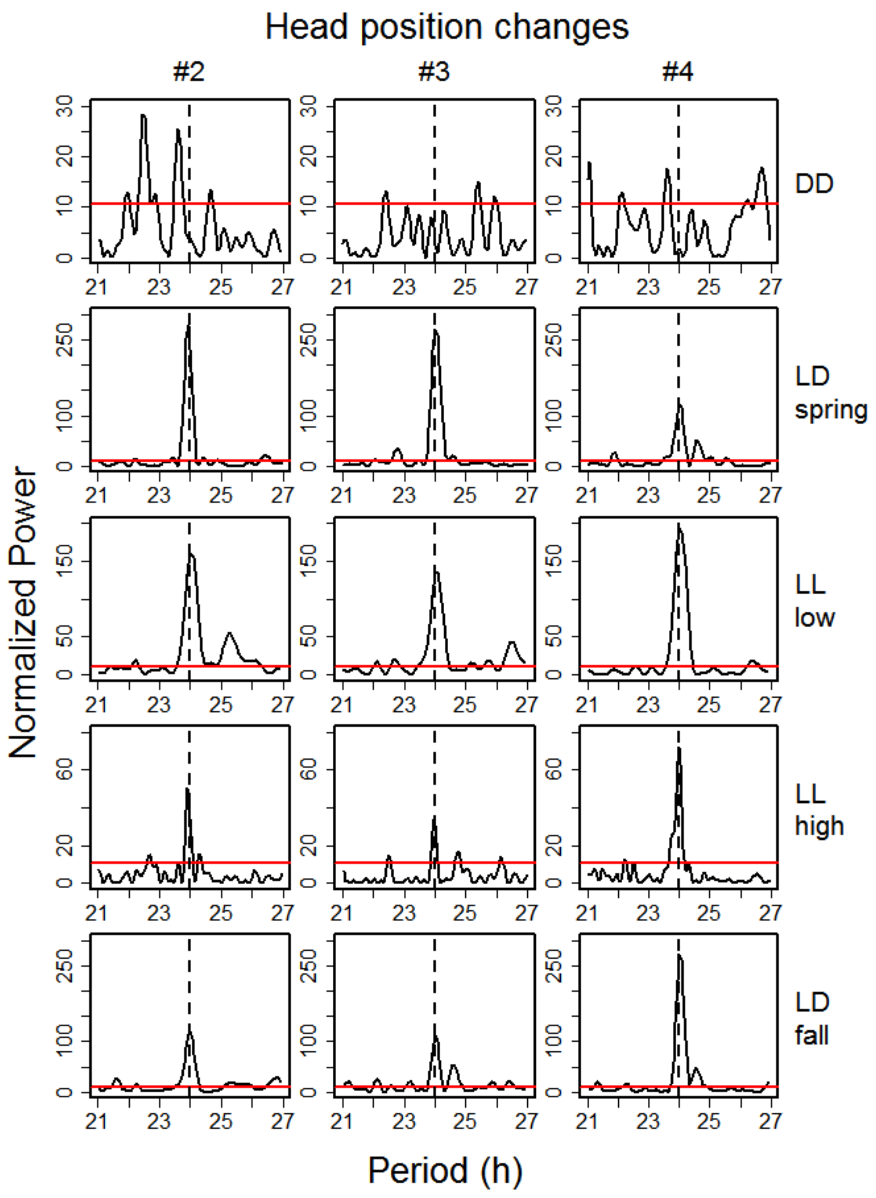

Figure S1 Comparison of rhythms of head position changes with period lengths (τ) between 21 and 27 hours of study animals #2, #3, and #4 between Nov 15, 2012 and Nov 15, 2013. Plotted are normalized powers of rhythms of activity. Each row of panels represents the different time periods: Polar Night (DD), daylight and night (LD), continual light (Midnight Sun, LL) before the onset of new vegetation growth with NDVI values <0 (LL low), and continual light with above-ground new vascular plant growth (NDVI>0, LL high). Horizontal red lines indicate the significance threshold of p=0.001.

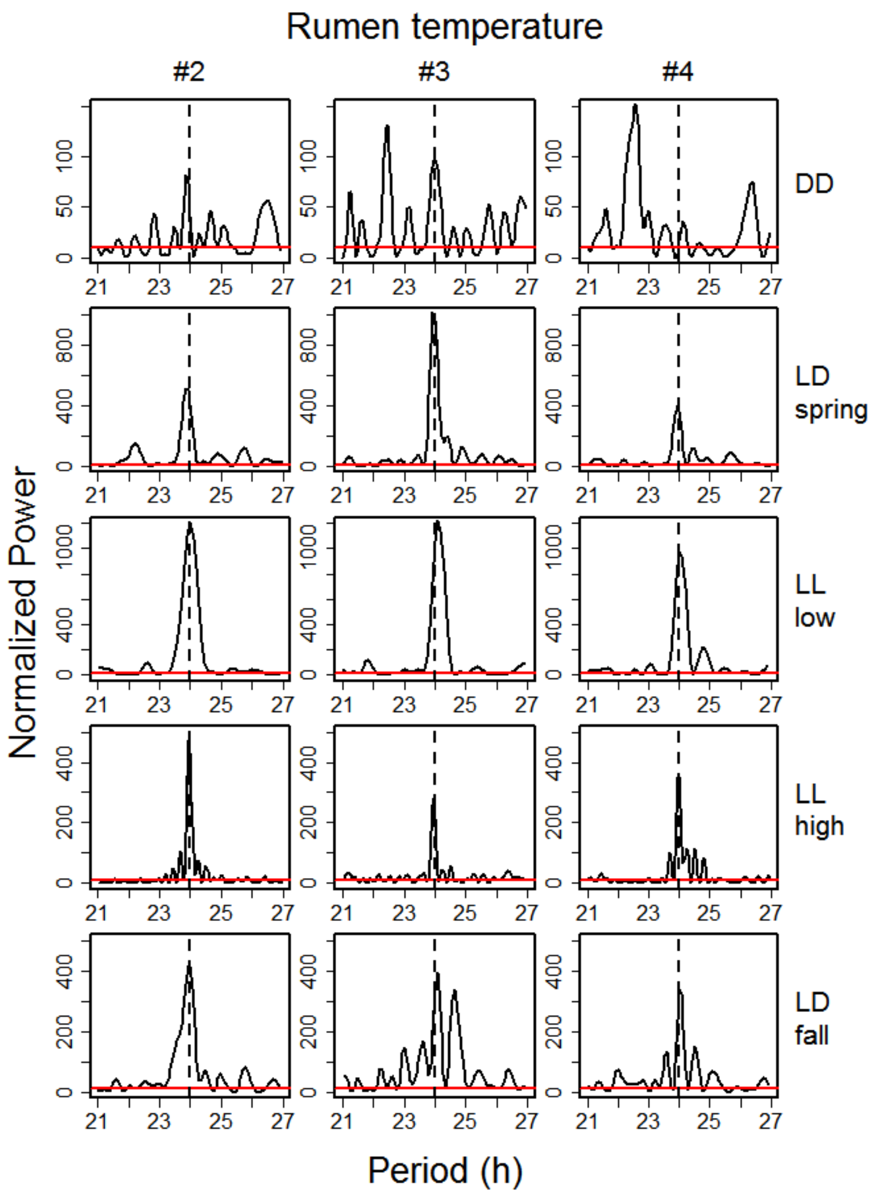

Figure S2 Comparison of rhythms of rumen temperature with period lengths (τ) between 21 and 27 hours of study animals #2, #3, and #4 between Nov 15, 2012 and Nov 15, 2013. Plotted are normalized powers of rhythms of activity. Each row of panels represents the different time periods: Polar Night (DD), daylight and night (LD), continual light (Midnight Sun, LL) before the onset of new vegetation growth with NDVI values <0 (LL low), and continual light with above-ground new vascular plant growth (NDVI>0, LL high). Horizontal red lines indicate the significance threshold of p=0.001.

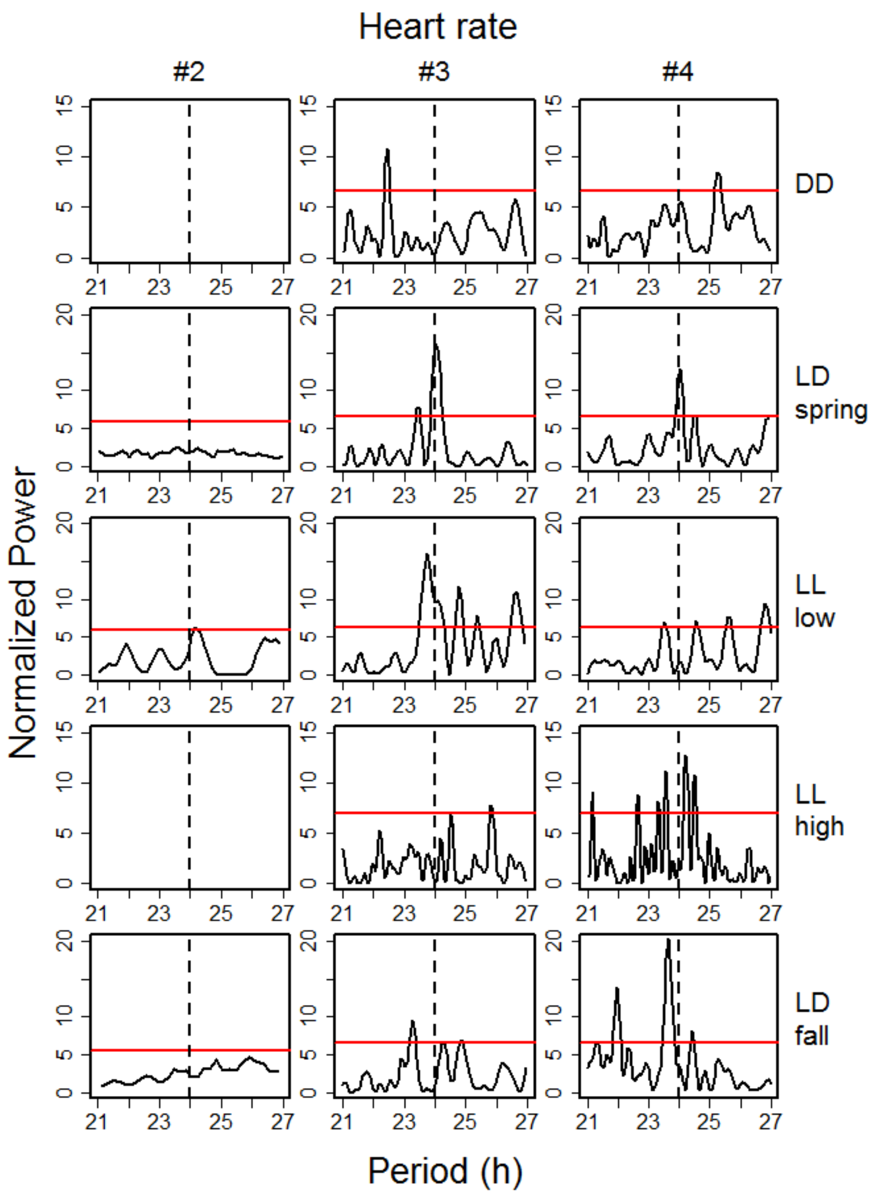

Figure S3 Comparison of rhythms of heart rate with period lengths (τ) between 21 and 27 hours of study animals #2, #3, and #4 between Nov 15, 2012 and Nov 15, 2013. Plotted are normalized powers of rhythms of activity. Each row of panels represents the different time periods: Polar Night (DD), daylight and night (LD), continual light (Midnight Sun, LL) before the onset of new vegetation growth with NDVI values <0 (LL low), and continual light with above-ground new vascular plant growth (NDVI>0, LL high). Horizontal red lines indicate the significance threshold of p=0.05.

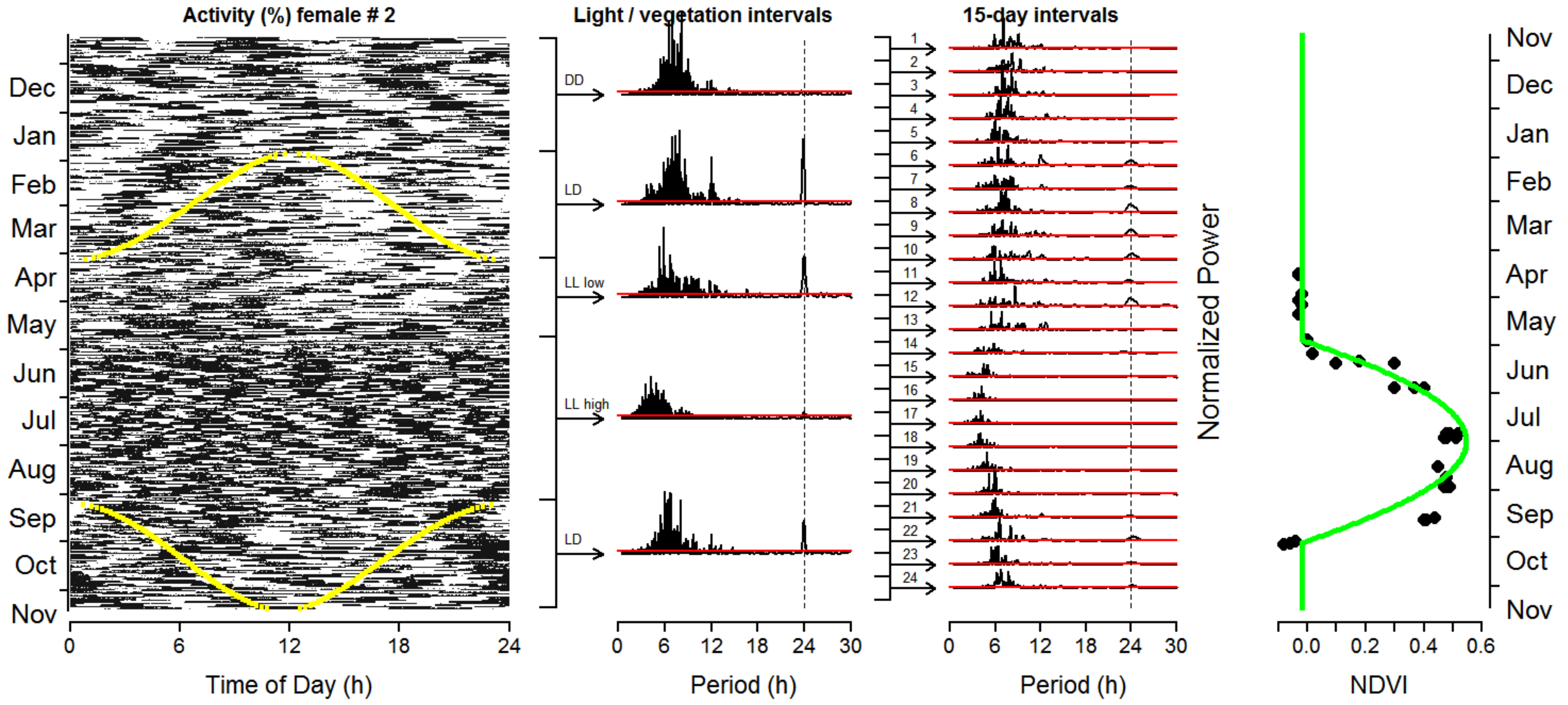

Figure S4 24 hour patterns of activity per 3-minute measurement interval and changes between Nov 15, 2012 and Nov 15, 2013 of individual #2. Panels from left to right: Actogram, yellow lines indicate beginning and end of civil twilight; periodograms for periods with different photic/vegetation conditions; periodograms for 15-day intervals; seasonal course of NDVI values. Horizontal red lines indicate the significance threshold of $p<0.001$. For details see legend to Fig. 5.

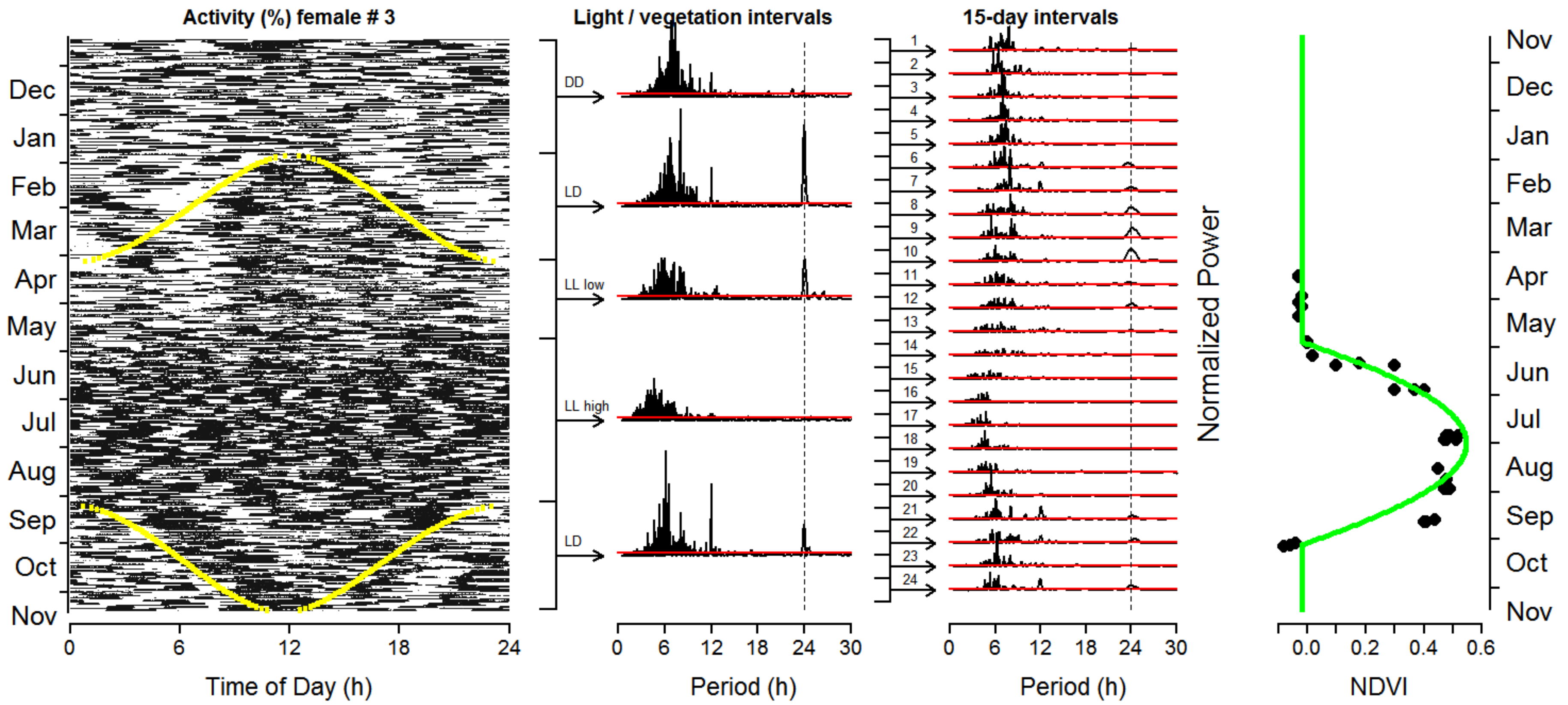

Figure S5 24 hour patterns of activity per 3-minute measurement interval and changes between Nov 15, 2012 and Nov 15, 2013 of individual #3. Panels from left to right: Actogram, yellow lines indicate beginning and end of civil twilight; periodograms for periods with different photic/vegetation conditions; periodograms for 15-day intervals; seasonal course of NDVI values. Horizontal red lines indicate the significance threshold of p<0.001. For details see legend to Fig. 5.

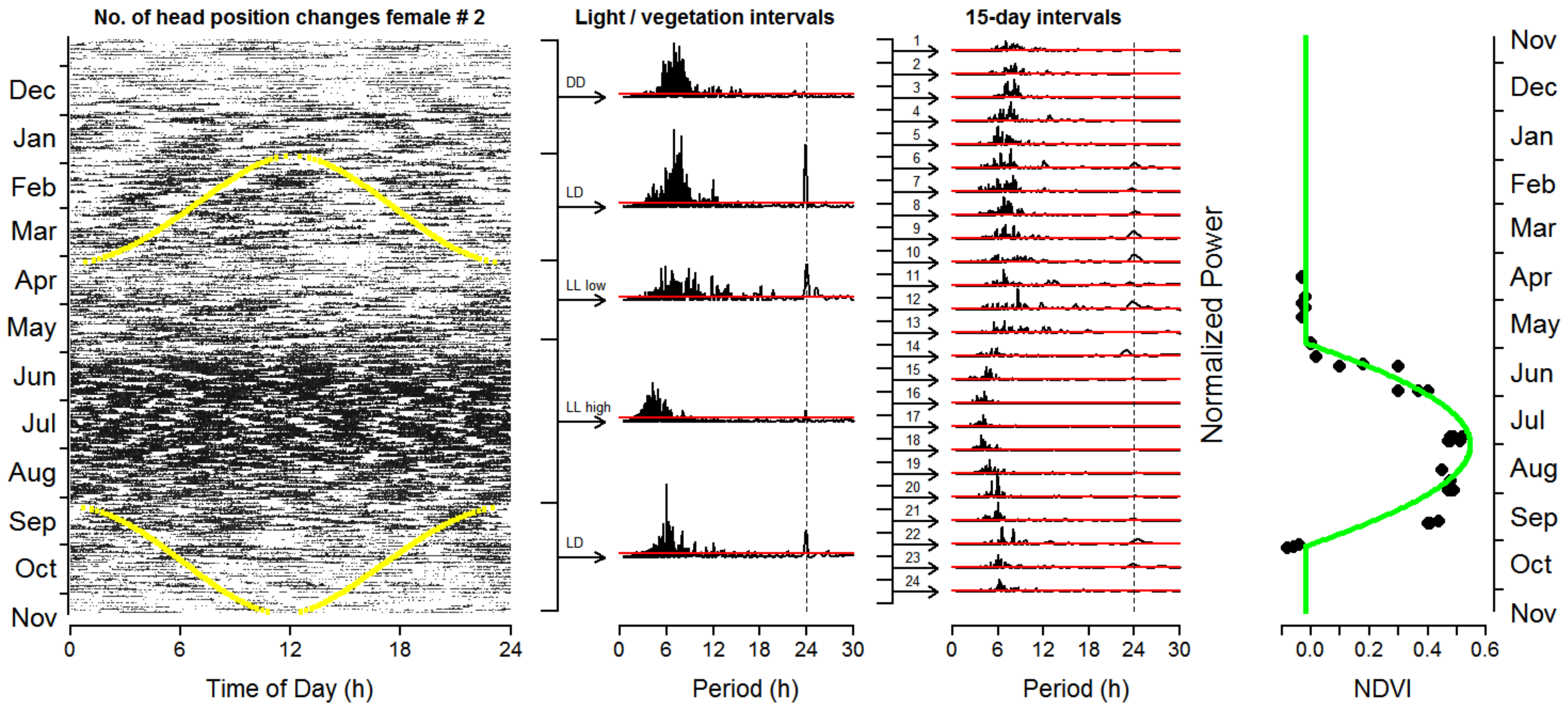

Figure S6 24 hour patterns of the number of head position changes per 3-minute measurement interval and changes between Nov 15, 2012 and Nov 15, 2013 of individual #2. Panels from left to right: Actogram, yellow lines indicate beginning and end of civil twilight; periodograms for periods with different photic/vegetation conditions; periodograms for 15-day intervals; seasonal course of NDVI values. Horizontal red lines indicate the significance threshold of p=0.001. For details see legend to Fig. 5.

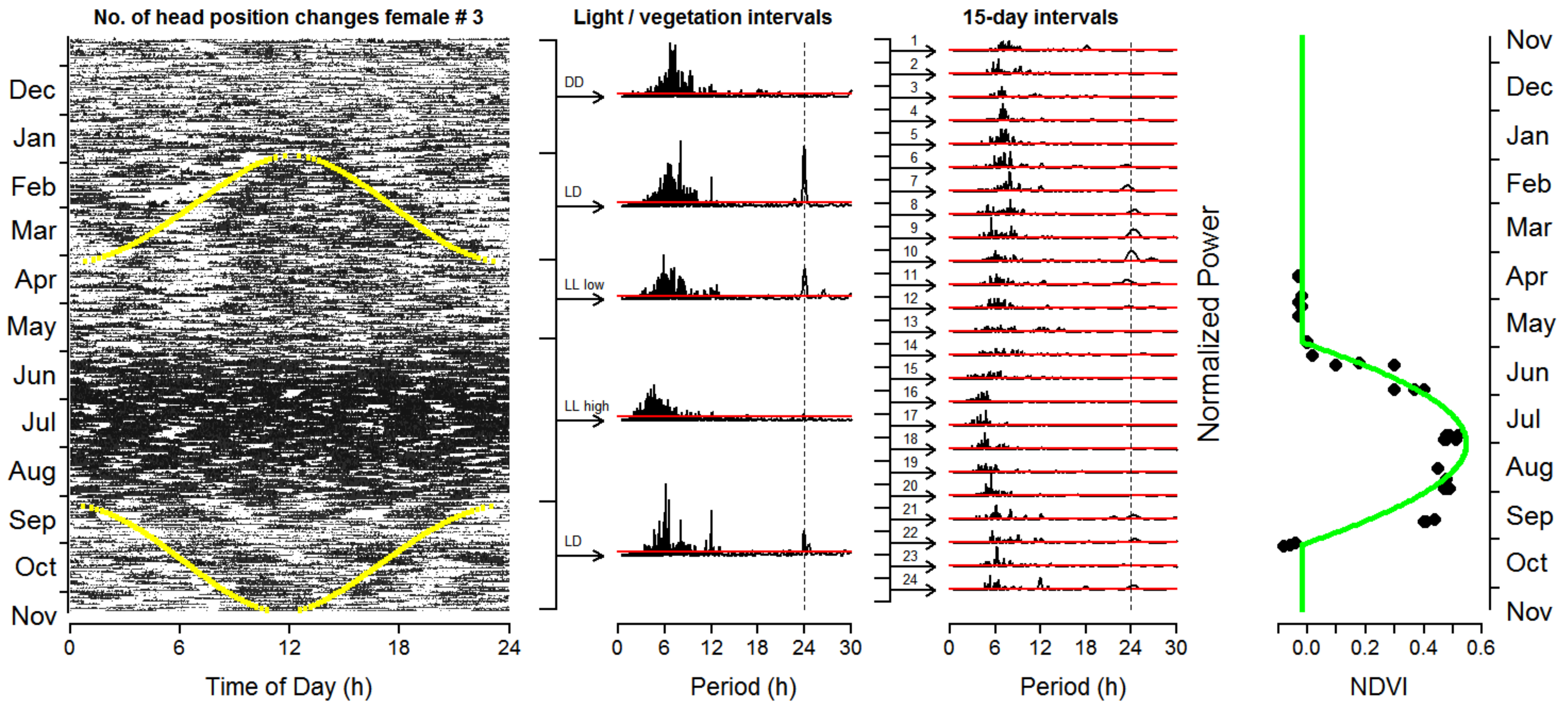

Figure S7 24 hour patterns of the number of head position changes per 3-minute measurement interval and changes between Nov 15, 2012 and Nov 15, 2013 of individual #3. Panels from left to right: Actogram, yellow lines indicate beginning and end of civil twilight; periodograms for periods with different photic/vegetation conditions; periodograms for 15-day intervals; seasonal course of NDVI values. Horizontal red lines indicate the significance threshold of p=0.001. For details see legend to Fig. 5.

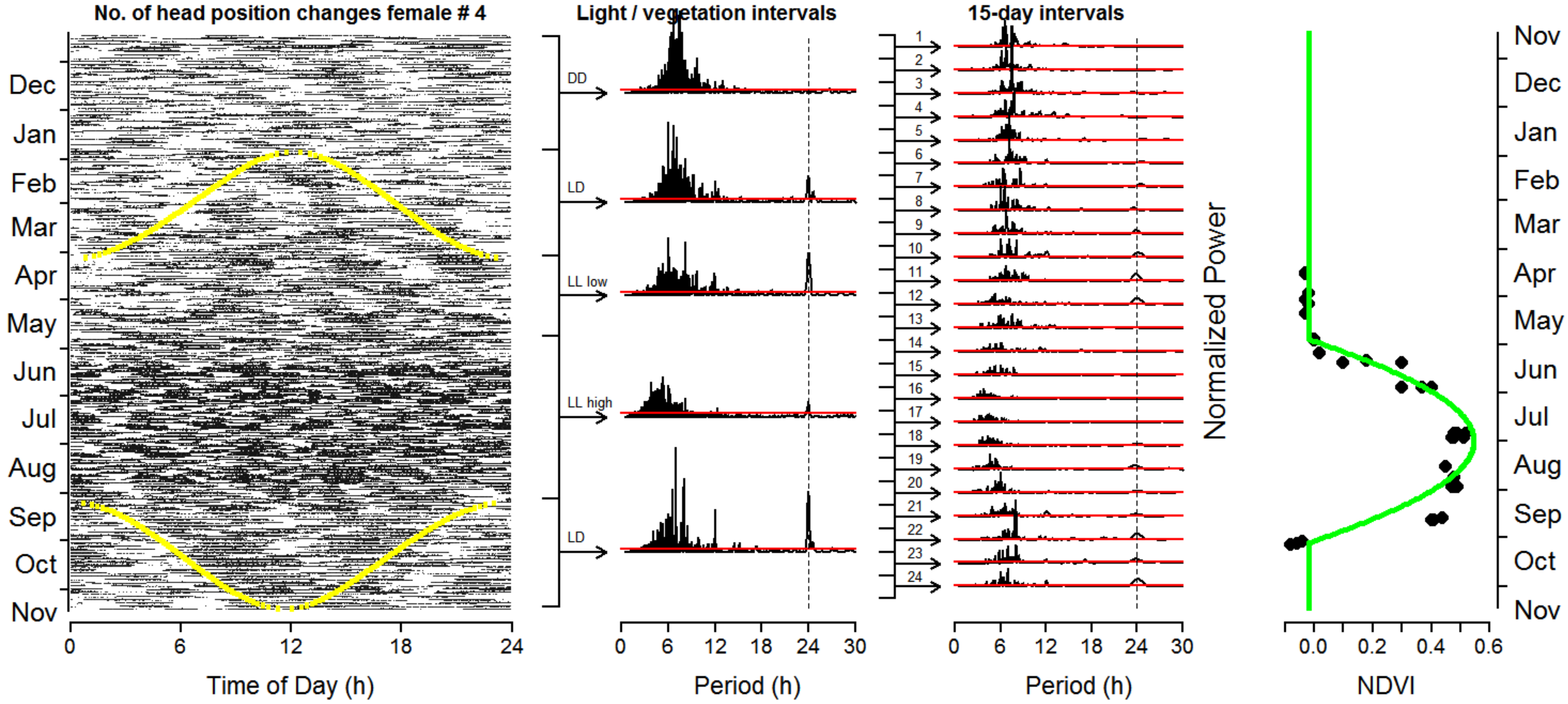

Figure S8 24 hour patterns of the number of head position changes per 3-minute measurement interval and changes between Nov 15, 2012 and Nov 15, 2013 of individual #4. Panels from left to right: Actogram, yellow lines indicate beginning and end of civil twilight; periodograms for periods with different photic/vegetation conditions; periodograms for 15-day intervals; seasonal course of NDVI values. Horizontal red lines indicate the significance threshold of p=0.001. For details see legend to Fig. 5.

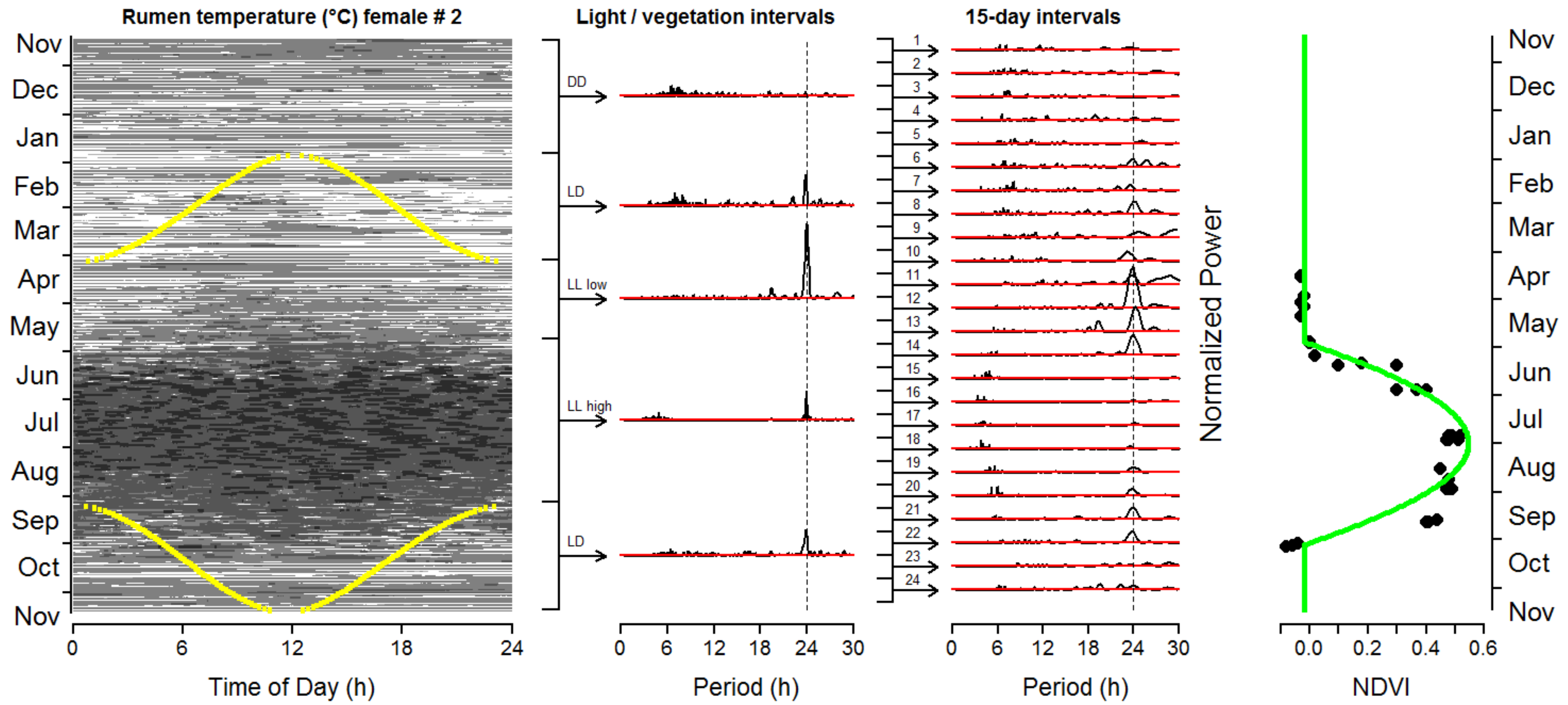

Figure S9 24 hour patterns of temperatures in the rumen, T_{rumen}, measured every 3-minutes and changes between Nov 15, 2012 and Nov 15, 2013 of individual #2. Panels from left to right: Actogram-like pattern of daily changes in T_{rumen}, with height of bars and their degree of darkness proportional to T_{rumen}, yellow lines indicate beginning and end of civil twilight; periodograms for periods with different photic/vegetation conditions; periodograms for 15-day intervals; seasonal course of NDVI values. Horizontal red lines indicate the significance threshold of p=0.001. For details see legend to Fig. 5.

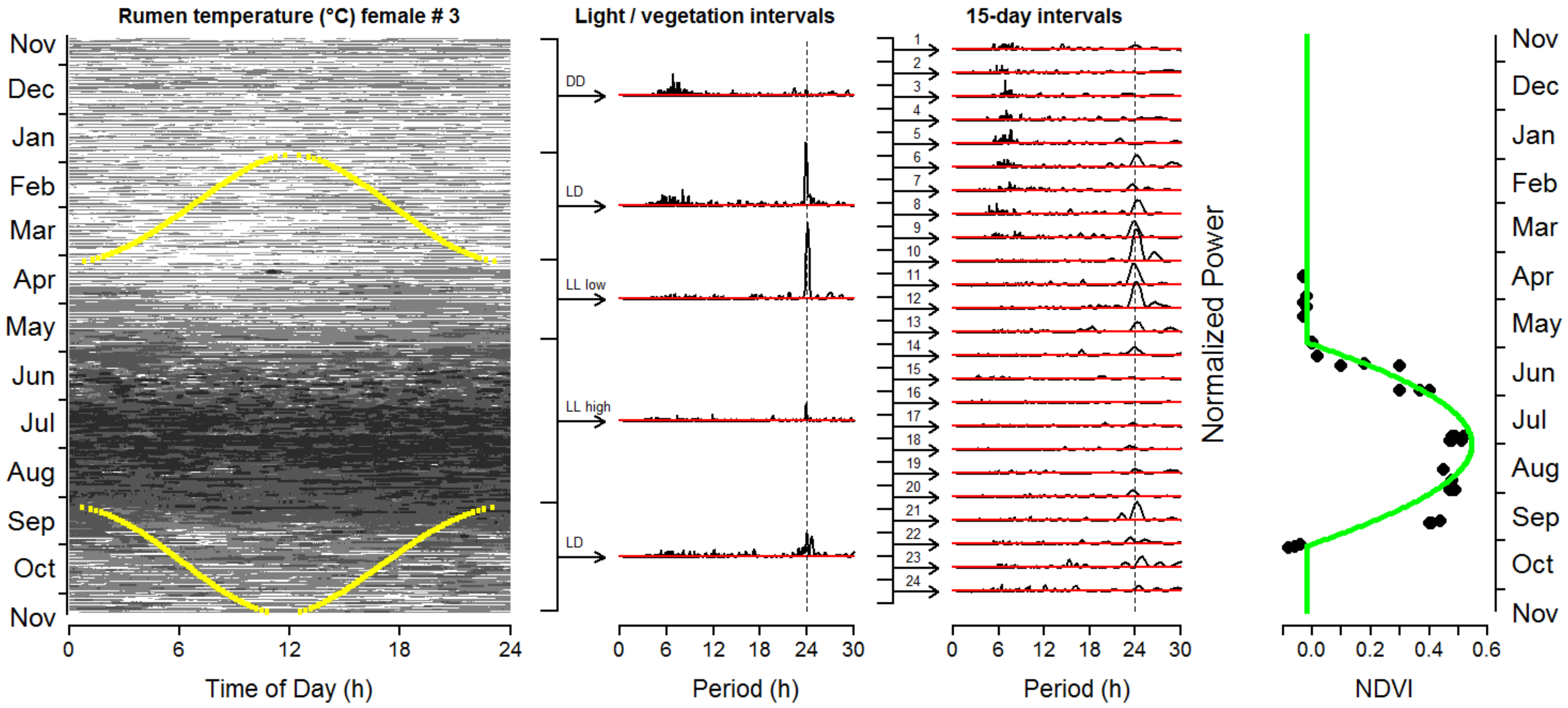

Figure S10 24 hour patterns of temperatures in the rumen, T_{rumen}, measured every 3-minutes and changes between Nov 15, 2012 and Nov 15, 2013 of individual #3. Panels from left to right: Actogram-like pattern of daily changes in T_{rumen}, with height of bars and their degree of darkness proportional to T_{rumen}, yellow lines indicate beginning and end of civil twilight; periodograms for periods with different photic/vegetation conditions; periodograms for 15-day intervals; seasonal course of NDVI values. Horizontal red lines indicate the significance threshold of p=0.001. For details see legend to Fig. 5.

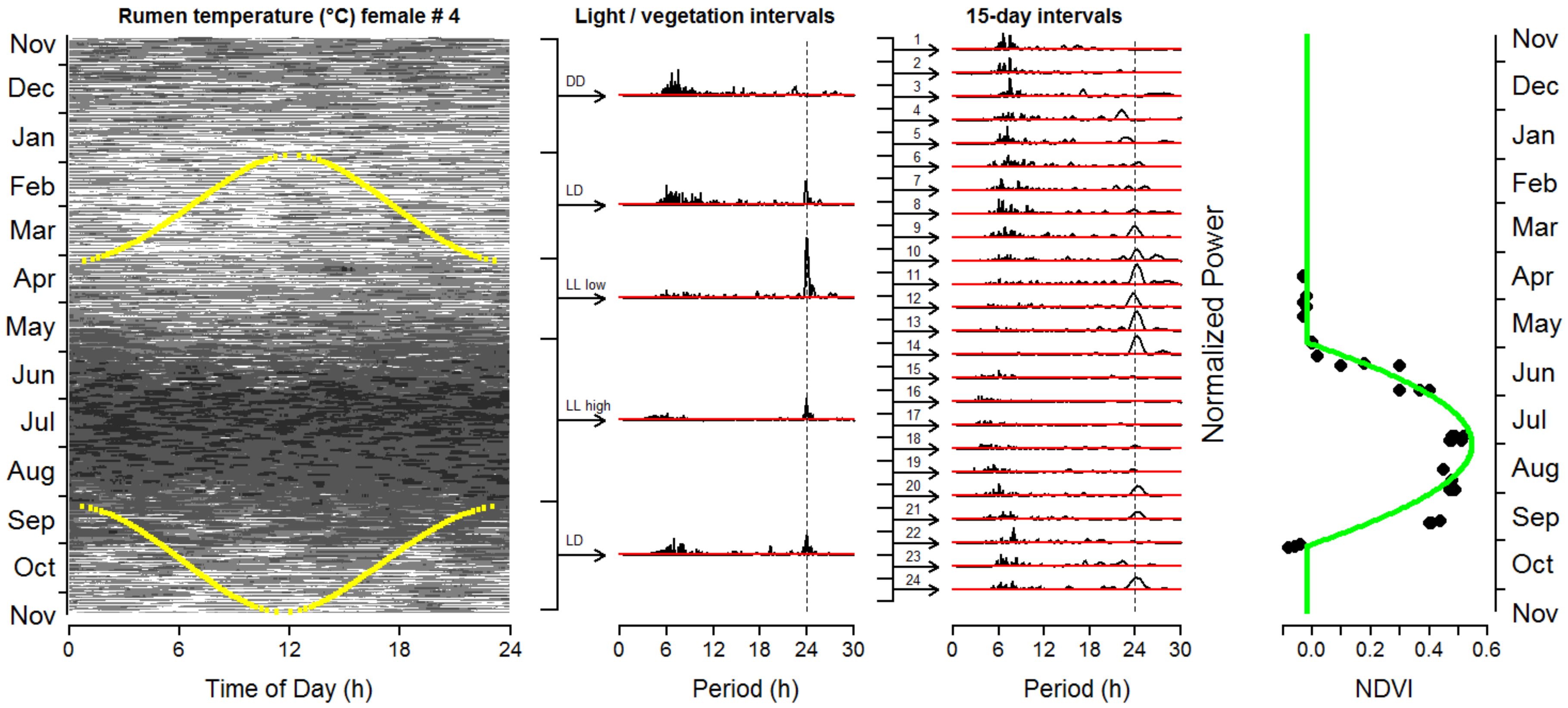

Figure S11 24 hour patterns of temperatures in the rumen, T_{rumen}, measured every 3-minutes and changes between Nov 15, 2012 and Nov 15, 2013 of individual #4. Panels from left to right: Actogram-like pattern of daily changes in T_{rumen}, with height of bars and their degree of darkness proportional to T_{rumen}, yellow lines indicate beginning and end of civil twilight; periodograms for periods with different photic/vegetation conditions; periodograms for 15-day intervals; seasonal course of NDVI values. Horizontal red lines indicate the significance threshold of p=0.001. For details see legend to Fig. 5.

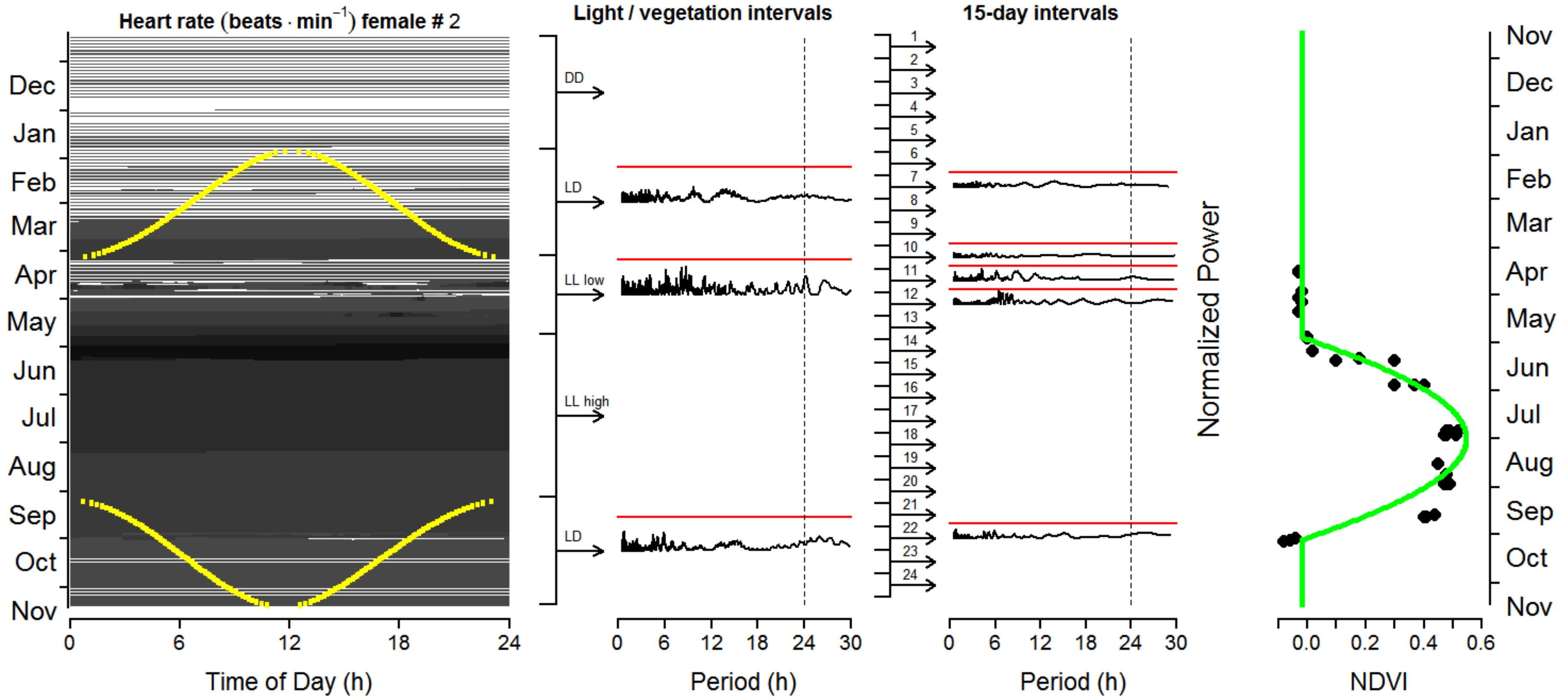

Figure S12 24 hour patterns of stationary heart rate, HR_s, measured every 21 minutes during a 3-minute interval, and changes between Nov 15, 2012 and Nov 15, 2013 of individual #2. Panels from left to right: Actogram-like pattern of daily changes in HR_s, with height of bars and their degree of darkness proportional to HR_s, yellow lines indicate beginning and end of civil twilight; periodograms for periods with different photic/vegetation conditions; periodograms for 15-day intervals, missing lines indicate insufficient sample size for Lomb-Scargle periodogram analysis; seasonal course of NDVI values. Horizontal red lines indicate the significance threshold of p=0.05. For details see legend to Fig. 5.

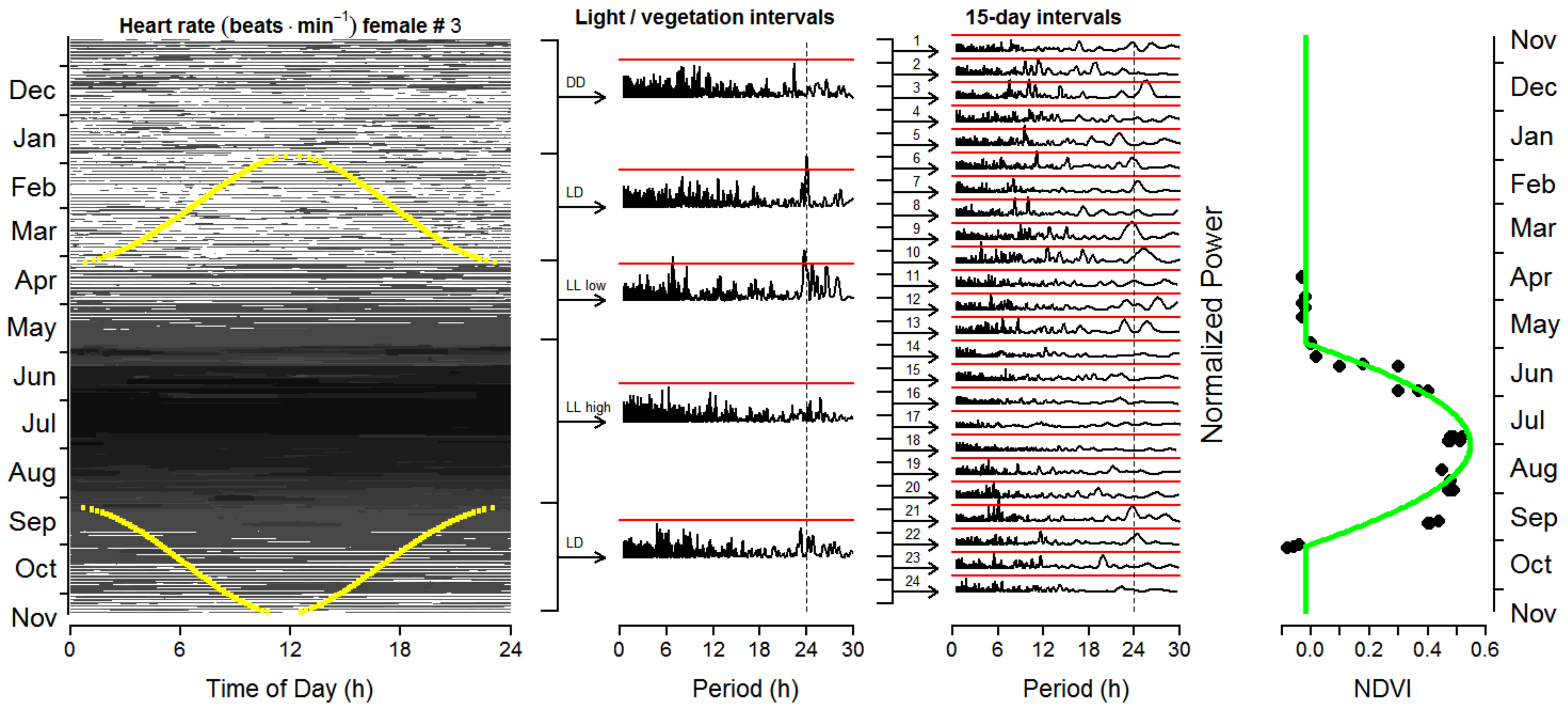

Figure S13 24 hour patterns of stationary heart rate, HR_s, measured every 21 minutes during a 3-minute interval, and changes between Nov 15, 2012 and Nov 15, 2013 of individual #3. Panels from left to right: Actogram-like pattern of daily changes in HR_s, with height of bars and their degree of darkness proportional to HR_s, yellow lines indicate beginning and end of civil twilight; periodograms for periods with different photic/vegetation conditions; periodograms for 15-day intervals, missing lines indicate insufficient sample size for Lomb-Scargle periodogram analysis; seasonal course of NDVI values. Horizontal red lines indicate the significance threshold of p=0.05. For details see legend to Fig. 5.

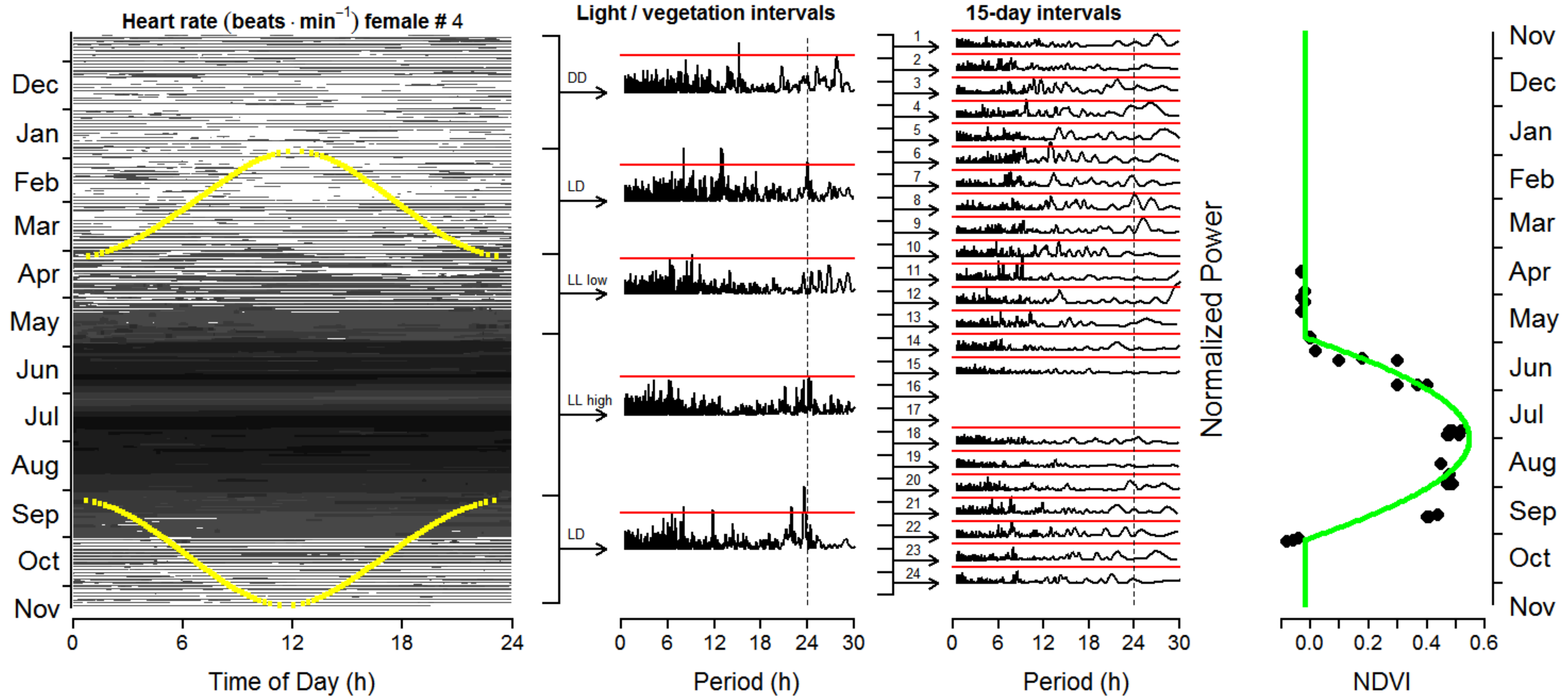

Figure S14 24 hour patterns of stationary heart rate, HR_s, measured every 21 minutes during a 3-minute interval, and changes between Nov 15, 2012 and Nov 15, 2013 of individual #4. Panels from left to right: Actogram-like pattern of daily changes in HR_s, with height of bars and their degree of darkness proportional to HR_s, yellow lines indicate beginning and end of civil twilight; periodograms for periods with different photic/vegetation conditions; periodograms for 15-day intervals, missing lines indicate insufficient sample size for Lomb-Scargle periodogram analysis; seasonal course of NDVI values. Horizontal red lines indicate the significance threshold of p=0.05. For details see legend to Fig. 5.
